# Supplementary material for: Development and validation of automated computer-aided risk scores to predict in-hospital mortality for emergency medical admissions with COVID-19: a retrospective cohort development and validation study
Source: BMJ Open. 2022 Aug 30;12(8):e050274. doi: 10.1136/bmjopen-2021-050274 (PMC9437732; doi:10.1136/bmjopen-2021-050274)
Supplement: Author's manuscript [file bmjopen-2021-050274.draft_revisions.pdf]

BMJ Open

BMJ Open is committed to open peer review. As part of this commitment we make the peer review history of every article we publish publicly available.

When an article is published we post the peer reviewers' comments and the authors' responses online. We also post the versions of the paper that were used during peer review. These are the versions that the peer review comments apply to.

The versions of the paper that follow are the versions that were submitted during the peer review process. They are not the versions of record or the final published versions. They should not be cited or distributed as the published version of this manuscript.

BMJ Open is an open access journal and the full, final, typeset and author-corrected version of record of the manuscript is available on our site with no access controls, subscription charges or pay-per-view fees (http://bmjopen.bmj.com).

If you have any questions on BMJ Open's open peer review process please email info.bmjopen@bmj.com

BMJ Open

Development and validation of automated computer aided-risk score to predict in-hospital mortality for COVID-19 hospital admissions: a retrospective cohort development and validation study

Journal:	*BMJ Open*
Manuscript ID	bmjopen-2021-050274
Article Type:	Original research
Date Submitted by the Author:	16-Feb-2021
Complete List of Authors:	Faisal, Muhammad; University of Bradford Faculty of Health Studies; Bradford Institute for Health Research<br>Mohammed, Mohammed ; University of Bradford Faculty of Health Studies; NHS Midlands and Lancashire Commissioning Support Unit<br>Richardson, Donald; York Teaching Hospital NHS Foundation Trust<br>Fiori, Massimo ; York Teaching Hospital NHS Foundation Trust<br>Beatson, Kevin; York Teaching Hospital NHS Foundation Trust
Keywords:	COVID-19, Infection control < INFECTIOUS DISEASES, Health & safety < HEALTH SERVICES ADMINISTRATION & MANAGEMENT, Quality in health care < HEALTH SERVICES ADMINISTRATION & MANAGEMENT

SCHOLARONE™
Manuscripts

I, the Submitting Author has the right to grant and does grant on behalf of all authors of the Work (as defined in the below author licence), an exclusive licence and/or a non-exclusive licence for contributions from authors who are: i) UK Crown employees; ii) where BMJ has agreed a CC-BY licence shall apply, and/or iii) in accordance with the terms applicable for US Federal Government officers or employees acting as part of their official duties; on a worldwide, perpetual, irrevocable, royalty-free basis to BMJ Publishing Group Ltd ("BMJ") its licensees and where the relevant Journal is co-owned by BMJ to the co-owners of the Journal, to publish the Work in this journal and any other BMJ products and to exploit all rights, as set out in our licence.

The Submitting Author accepts and understands that any supply made under these terms is made by BMJ to the Submitting Author unless you are acting as an employee on behalf of your employer or a postgraduate student of an affiliated institution which is paying any applicable article publishing charge ("APC") for Open Access articles. Where the Submitting Author wishes to make the Work available on an Open Access basis (and intends to pay the relevant APC), the terms of reuse of such Open Access shall be governed by a Creative Commons licence – details of these licences and which Creative Commons licence will apply to this Work are set out in our licence referred to above.

Other than as permitted in any relevant BMJ Author's Self Archiving Policies, I confirm this Work has not been accepted for publication elsewhere, is not being considered for publication elsewhere and does not duplicate material already published. I confirm all authors consent to publication of this Work and authorise the granting of this licence.

For peer review only

Development and validation of automated computer aided-risk score to predict in-hospital mortality for COVID-19 hospital admissions: a retrospective cohort development and validation study

Authors

Muhammad Faisal, PhD
Senior Research Fellow in Biostatistics
Faculty of Health Studies, University of Bradford, Bradford, UK
Bradford Institute for Health Research Bradford, UK
NIHR Yorkshire and Humber Patient Safety Translational Research Centre (YHPSTRC), Bradford, UK
Wolfson Centre for Applied Health Research, Bradford, UK
E-mail: M.Faisal1@bradford.ac.uk

Mohammed A Mohammed, PhD
Professor of Healthcare Quality & Effectiveness
Faculty of Health Studies, University of Bradford, Bradford, UK
NHS Midlands and Lancashire Commissioning Support Unit,
The Strategy Unit,
Kingston House,
West Bromwich, B70 9LD, UK
E-mail: M.A.Mohammed5@Bradford.ac.uk

Donald Richardson, FRCP
Deputy Medical Director , Chief Clinical Information Officer, Consultant Renal Physician
Department of Renal Medicine, York Teaching Hospitals NHS Foundation Trust
E-mail: drichardson@doctors.org.uk

Massimo Fiori
Senior Analyst/ Programmer
York Teaching Hospitals NHS Foundation Trust, England UK
E-mail: massimo.fiori@york.nhs.uk

Kevin Beatson, MSc
Development Manager
York Teaching Hospitals NHS Foundation Trust, England UK
E-mail: Kevin.Beatson@York.NHS.uk

Correspondence to: M A Mohammed

For peer review only

Abstract

Objectives: There are no established mortality risk equations specifically for unplanned emergency medical admissions which include patients with the novel coronavirus SARS-19(COVID-19). We aim to develop and validate a computer-aided risk score (CARMc19) for predicting mortality risk by combining COVID-19 status, the first electronically recorded blood test results and the National Early Warning Score (NEWS2).

Design: Logistic regression model development and validation study

Setting: Two acute hospitals (YH – model development data; SH – external validation data).

Participants: Adult (>=16 years) medical admissions discharged over a 24 month period with electronic NEWS and blood test results recorded on admission. We used logistic regression modelling to predict the risk of in-hospital mortality using two models: 1) CARMc19_N: age + sex + NEWS2 including subcomponents + COVID19; 2) CARMc19_NB: CARMc19_N in conjunction with seven blood test results and acute kidney injury score. Model performance was evaluated according to discrimination (c-statistic), calibration (graphically), and clinical usefulness at NEWS2 thresholds of 4+, 5+, 6+.

Results: The risk of in-hospital mortality following emergency medical admission was similar in development and validation datasets (8.4% vs 8.2%). The c-statistics for predicting mortality for CARMc19_NB is better than CARMc19_N in the validation dataset (CARMc19_NB=0.88(95%CI 0.86-0.90) vs CARMc19_N=0.86(95%CI 0.83-0.88)). Both models had good calibration (CARMc19_NB=1.01(95%CI 0.88-1.14) & CARMc19_N:0.95(95%CI 0.83-1.06)). At all NEWS2 thresholds (4+, 5+, 6+) model CARMc19_NB had better sensitivity and similar specificity.

Conclusions: We have developed a validated CARMc19 scores with good performance characteristics for predicting the risk of in-hospital mortality. Since the CARMc19 scores place no additional data collection burden on clinicians, it may now be carefully introduced and evaluated in hospitals with sufficient informatics infrastructure.

Keywords: vital signs, national early warning score, emergency admission, novel coronavirus SARS-19, computer-aided national early warning score

For peer review only

Article Summary

- Whilst there are no established mortality risk equations specifically for unplanned emergency medical admissions which include patients with the novel coronavirus SARS-19(COVID-19), we aim to develop and validate a computer-aided risk score (CARMc19) for predicting mortality risk
- CARMc19 scores had good discrimination and calibration for predicting the risk of in-hospital mortality.
- Since the CARMc19 scores place no additional data collection burden on clinicians, it may now be carefully introduced and evaluated in hospitals with sufficient informatics infrastructure.

For peer review only

Introduction

The novel coronavirus SARS-19 produces the newly identified disease 'COVID-19' in patients with symptoms (Coronaviridae Study Group of the International Committee on Taxonomy of Viruses[1]) and was declared a pandemic on 11 March 2020 that has challenged health care systems worldwide.

COVID-19 patients admitted to hospital can develop severe disease with life threatening respiratory and/or multi-organ failure [2,3] with a high risk of mortality. The appropriate early assessment and management of patients with COVID-19 is important in ensuring high-quality care including isolation, escalation to critical care or palliative care.

Early Warning Scores (EWS) are commonly used in hospitals worldwide [4], and in the National Health Service (NHS) hospitals in England, the patient's vital signs are monitored and summarised into a National Early Warning Score (NEWS)[5]. We have developed two automated risk equations to predict the patient's risk of mortality (CARM_N & CARM_NB) using NEWS only (CARM_N) [6] and NEWS + blood test results (CARM_NB) [7] following emergency medical admission to hospital. We found CARM performed similar to consultant clinicians [8].

In December 2017, an update to NEWS (NEWS2) was published [4] that extends the level of consciousness from AVPU to ACVPU, where C represents new confusion or delirium and is allocated three points (the maximum for a single variable). NEWS2 also offers two scales for oxygen saturation (scale 1 and scale 2) which accommodates patients with hypercapnic respiratory failure who have clinically recommended oxygen saturation of 88–92%.

Whilst hospitals continue to use NEWS2 during the COVID-19 pandemic, little is known about how NEWS2 and CARM scores perform in monitoring COVID-19 patients. In this study, we aimed to develop and validate an automated computer aided risk score (CARMc19) using on admission NEWS2 and blood test results for predicting mortality. This approach is clinically useful because it places no additional data collection burden on staff for monitoring COVID-19 patients.

For peer review only

Methods

Setting & data

Our cohorts of emergency medical admissions are from two acute hospitals which are approximately 65 kilometres apart in the Yorkshire & Humberside region of England – Scarborough hospital (n~300 beds) and York Hospital (YH) (n~700 beds), managed by York Teaching Hospitals NHS Foundation Trust. We selected these hospitals because they had electronic NEWS2, collected as part of the patient's process of care since April 2019, and were agreeable to the study.

We considered all consecutive adult (age≥18 years) non-elective or emergency medical admissions discharged over a course of three months (11 March 2020 to 13 June 2020) with electronic NEWS2. For each emergency admission, we obtained a pseudonymised patient identifier, patient's age (years), sex (male/female), discharge status (alive/dead), admission and discharge date and time, diagnoses codes based on the 10th revision of the International Statistical Classification of Diseases (ICD-10), NEWS2 (including its subcomponents respiratory rate, temperature, systolic pressure, pulse rate, oxygen saturation, oxygen supplementation, oxygen scales 1 & 2, and alertness including confusion), blood test results (albumin, creatinine, haemoglobin, potassium, sodium, urea, and white cell count), and Acute Kidney Injury (AKI) score.

The diastolic blood pressure was recorded at the same time as systolic blood pressure. Historically, diastolic blood pressure has always been a routinely collected physiological variable on vital sign charts and is still collected where electronic observations are in place. NEWS2 produces integer values that range from 0 (indicating the lowest severity of illness) to 20 (the maximum NEWS2 value possible) (see Table S1 in supplementary material). The index NEWS2 was defined as the first electronically recorded NEWS2 within ±24 hours of the admission time. We excluded records where the index NEWS2 (or blood test results) was not within ±24 hours (±96 hours) or was missing/not recorded at all (see Table S2). The ICD-10 code 'U071' was used to identify records with COVID-19. We searched primary and secondary ICD-10 codes for 'U071' for identifying COVID-19.

Statistical Modelling

We began with exploratory analyses including box plots and line plots to show the relationship between covariates and risk of in-hospital mortality. We developed two logistic regression models, known as CARMc19_N and CARMc19_NB, to predict the risk of in-hospital mortality with following covariates: 1) Model CARMc19_N uses age + sex + COVID-19 (yes/no) + NEWS2 including sub components; 2) Model CARMc19_NB extends Model CARMc19_N with all seven blood test results and AKI score. The primary rationale for using these variables is that they are routinely collected as part of

For peer review only

process of care and their inclusion in our statistical models is on clinical grounds as opposed to the statistical significance of any given covariate.

We used the *qladder* function (Stata [9]), which displays the quantiles of a transformed variable against the quantiles of a normal distribution according to the ladder powers $(x^3, x^2, x^1, x, \sqrt{x}, \log(x), x^{-1}, x^{-2}, x^{-3})$ for each continuous covariate and chose the following transformations: (creatinine)-1/2, $\log_e$(potassium), $\log_e$(white cell count), $\log_e$(urea), $\log_e$ (respiratory rate), $\log_e$(pulse rate), $\log_e$(systolic blood pressure), and $\log_e$(diastolic blood pressure). We used an automated approach to search for all two-way interactions and incorporated those interactions which were statistically significant ($p<0.001$) from the MASS library [12] in *R* [13].

We developed both models using York Hospital (YH) data (development dataset) and externally validated their performance on Scarborough Hospital (SH) data (validation dataset). The hospitals are part of the same NHS Trust but are geographically separated by about 65 kilometres (40 miles).

We report discrimination and calibration statistics as performance measures for these models [10].

Discrimination relates to how well a model can separate – or discriminate between – those who died and those who did not and is given by the area under the Receiver Operating Characteristics (ROC) curve (AUC) or c-statistic. The ROC curve is a plot of the sensitivity (true positive rate) versus 1-specificity (false positive rate) for consecutive predicted risks. A c-statistic of 0.5 is no better than tossing a coin, whilst a perfect model has a c-statistic of 1. In general, values less than 0.7 are considered to show poor discrimination, values of 0.7 to 0.8 can be described as reasonable, and values above 0.8 suggest good discrimination [11]. The 95% confidence interval for the c-statistic was derived using DeLong's method as implemented in the *pROC* library [12] in *R* [13].

Calibration measures a model's ability to generate predictions that are, on average, close to the average observed outcome and can be readily seen on a scatter plot (y-axis = observed risk, x-axis = predicted risk). Perfect predictions should be on the 45° line. We internally validated and assessed the calibration for all the models using the bootstrapping approach [14,15]. The overall statistical performance was assessed using the scaled Brier score which incorporates both discrimination and calibration [10]. The Brier score is the squared difference between actual outcomes and predicted risk of death, scaled by the maximum Brier score such that the scaled Brier score ranges from 0–100%. Higher values indicate superior models.

The recommended threshold for detecting deteriorating patients and sepsis is NEWS2≥5 [16,17]. Therefore, we assessed the sensitivity, specificity, positive and negative predictive values and likelihood ratios for these models at NEWS2 threshold of 4+,5+, and 6+ [18]. We followed the TRIPOD

For peer review only

guidelines for reporting of model development and validation [19]. We used Stata [9] for data cleaning and *R* [13] for statistical analysis.

Results

Cohort Characteristics

The number of non-elective discharges was 6444 over 3 months. For the development of CARMc19_N, we excluded 36 (0.6%) of admissions because the index NEWS2 was not recorded within ±24 hours of the admission date/time, or they were missing or not recorded at all (see Table S2). Likewise, for the development of CARMc19_NB, we further excluded 1189 (18.3%) of admissions because the first blood test results were not recorded within ±96 hours of the admission date/time, or they were missing or not recorded at all (see Table S2).

The characteristics of the admissions included in our study are shown in Table 1. Emergency admissions in the validation dataset were older than those in development dataset (69.6 years vs 67.4 years), less likely to be male (49.5% vs 51.2%), had higher index NEWS2 (3.2 vs 2.8), higher prevalence of COVID-19 (11.0% vs 8.7%) but similar in-hospital mortality (8.4% vs 8.2%). See accompanying scatter and boxplots in Figure S1 to S4 (supplemental digital content).

For peer review only

Characteristic	Development dataset (YH)	Validation dataset (SH)	Degree of freedom (df)	p-value
N	3924	2520		-
Male (%)	2010 (51.2)	1247 (49.5)	1	0.181
Mean Age [years] (SD)	67.4 (18.7)	69.6 (18.9)	5320	<0.001
Median Length of Stay (days) (IQR)	3.0 (5.8)	3.7 (6.1)	-	<0.001
COVID-19 (%)	343 (8.7)	277 (11.0)	1	0.003
**Mortality**				
Mortality with-in 24 hours (%)	30 (0.8)	32 (1.3)	1	0.058
Mortality with-in 48 hours (%)	61 (1.6)	48 (1.9)	1	0.335
Mortality with-in 72 hours (%)	96 (2.4)	68 (2.7)	1	0.585
In-hospital mortality	323 (8.2)	212 (8.4)	1	0.833
Mean NEWS2 (SD)	2.8 (2.8)	3.2 (2.8)	5446	<0.001
**Vital Signs**				
Mean Respiratory rate [breaths per minute] (SD)	19.8 (5.1)	20.7 (5.6)	5027	<0.001
Mean Temperature [^{o}C] (SD)	36.4 (0.9)	36.3 (1)	4817	0.001
Mean Systolic pressure [mmHg] (SD)	141.8 (29.2)	142 (28.5)	5455	0.839
Mean Diastolic pressure [mmHg] (SD)	79.2 (16.5)	79 (17.3)	5193	0.545
Mean Pulse rate [beats per minute] (SD)	89.1 (22.3)	88.5 (22.1)	5406	0.336
Mean Oxygen saturation (SD)	96.3 (3.1)	96.1 (3.2)	5182	0.059
Oxygen supplementation (%)	512 (13)	362 (14.4)	1	0.142
Mean oxygen flow rate (units) (SD)	7.1 (5.7)	6.1 (5.3)	811	0.007
Oxygen scale 2 (yes) (%)	240 (6.1)	163 (6.5)	1	0.605
**Alertness**				
Alert (%)	3510 (89.4)	2243 (89)	5	0.010
Baseline confusion (%)	27 (0.7)	23 (0.9)		
New confusion (%)	61 (1.6)	40 (1.6)		
Pain (%)	32 (0.8)	17 (0.7)		
Voice (%)	151 (3.8)	134 (5.3)		
Unconscious (%)	143 (3.6)	63 (2.5)		
Mean Albumin [g/L] (SD)	40.3 (5.7)	40.2 (5.8)	4484	0.508
Mean Creatinine [umol/L] (SD)	106.3 (104.1)	103 (82.5)	5125	0.194
Mean Haemoglobin [g/l] (SD)	126.1 (23.4)	127.5 (22.3)	4680	0.027
Mean Potassium [mmol/L] (SD)	4.4 (0.6)	4.4 (0.6)	4449	0.135
Mean Sodium [mmol/L] (SD)	138.3 (5)	137.9 (5.3)	4349	0.016
Mean White cell count [10^9 cells/L] (SD)	10.3 (7.6)	11 (5.9)	5147	<0.001
Mean Urea [mmol/L] (SD)	7.9 (6.2)	8.3 (6.6)	4382	0.017
AKI Score			2.2	0.158
0 (%)	2900 (92)	1916 (90.5)		
1 (%)	137 (4.3)	120 (5.7)		
2 (%)	61 (1.9)	46 (2.2)		
3 (%)	53 (1.7)	36 (1.7)		

Table 1 Characteristics of emergency medical admissions in development and validation datasets.

We assessed the performance of both (CARMc19_N and CARMc19_NB) models to predict the risk of in-hospital mortality in emergency medical admissions (see Table 2 and Figure 1 for validation results and Table S3 and Figure S7 for model development results).

The c-statistics for predicting mortality for Model CARMc19_NB is better than Model CARMc19_N in development dataset (CARMc19_NB = 0.87 (95%CI 0.85 to 0.89) vs CARMc19_N = 0.86 (95%CI 0.84 to 0.87)) and the validation dataset (CARMc19_NB = 0.88 (95%CI 0.86 to 0.90) vs CARMc19_N = 0.86 (95%CI 0.83 to 0.88)).

Model	Mean risk discharged alive	Mean risk discharged deceased	ARD	Scaled brier score	AUC (95% CIs)	Calibration Slope
CARMc19_N	0.07	0.29	0.22	0.20	0.86<br>(0.83 to 0.88)	0.95<br>(0.83 to 1.06)
CARMc19_NB	0.07	0.30	0.23	0.22	0.88<br>(0.86 to 0.90)	1.01<br>(0.88 to 1.14)

Table 2: Performance of CARMc19_N and CARMc19_NB models for predicting the risk of mortality in validation dataset

ARD: Absolute risk difference; AUC: Area under the curve; CIs: Confidence intervals

Internal validation of both models is shown in Figure S6. Both models had good internal and external calibration (CARMc19_NB: 1.01 (95%CI 0.88 vs 1.14) & CARMc19_N: 0.95 (95%CI 0.83 to 1.06)) (see Table 2 and Figure 2).

Table 3 includes the sensitivity, specificity, positive and negative predictive values for CARMc19_N and CARMc19_NB models for predicting mortality at NEWS2 threshold of 4+, 5+, 6+.

At all NEWS2 thresholds (4+, 5+, 6+) model CARMc19_NB had better sensitivity (development dataset: 76% vs 72%; 71% vs 67%; 65% vs 61% and validation dataset: 79% vs 73%; 75% vs 68%; 69% vs 61%)

For peer review only

and similar specificity (development dataset: 81% vs 82%; 86% vs 86%; 89% vs 90% and validation dataset: 80% vs 82%; 85% vs 86%; 88% vs 89%) (see Table 3 & S4).

For peer review only

Model	At NEWS score [predicted risk of death]	Number of deaths identified by model	Sensitivity%	Specificity%	PPV	NPV	LR+	LR-
CARMc19_N	4+ [0.09]	696	73.1 (66.6 to 79)	81.8 (80.2 to 83.4)	27 (23.4 to 30.8)	97.1 (96.2 to 97.8)	4 (3.6 to 4.5)	0.3 (0.3 to 0.4)
CARMc19_N	5+ [0.11]	557	68.4 (61.7 to 74.6)	86 (84.5 to 87.4)	31 (26.8 to 35.4)	96.7 (95.9 to 97.5)	4.9 (4.3 to 5.6)	0.4 (0.3 to 0.4)
CARMc19_N	6+ [0.14]	452	61.8 (54.9 to 68.4)	89.1 (87.8 to 90.4)	34.3 (29.5 to 39.3)	96.2 (95.3 to 97)	5.7 (4.9 to 6.7)	0.4 (0.4 to 0.5)
CARMc19_NB	4+ [0.09]	651	79.1 (72.5 to 84.8)	79.8 (77.9 to 81.6)	26.9 (23.2 to 30.9)	97.6 (96.7 to 98.3)	3.9 (3.5 to 4.4)	0.3 (0.2 to 0.3)
CARMc19_NB	5+ [0.11]	526	75.3 (68.3 to 81.4)	84.8 (83.1 to 86.3)	31.7 (27.3 to 36.3)	97.3 (96.4 to 98)	4.9 (4.3 to 5.6)	0.3 (0.2 to 0.4)
CARMc19_NB	6+ [0.14]	431	69.2 (62 to 75.8)	88.1 (86.5 to 89.5)	35.3 (30.3 to 40.5)	96.8 (95.9 to 97.6)	5.8 (5 to 6.8)	0.3 (0.3 to 0.4)

Table 3 Sensitivity analysis of CARMc19_N and CARMc19_NB models in validation dataset for predicting the risk of mortality at NEWS2 thresholds 4+ [0.09] ,5+ [0.11], and 6+ [0.14] of predicted risk of mortality in development dataset.

PPV = Positive Predictive Value; NPV = Negative Predictive Value; LR+ = Positive Likelihood Ratio; LR- = Negative Likelihood Ratio

For peer review only

Discussion

In this study, we developed and validated two (CARMc19_N and CARMc19_NB) models to predict the risk of in-hospital mortality with the following covariates: 1) Model CARMc19_N uses age + sex + COVID-19 (yes/no) + NEWS2 including sub components; 2) Model CARMc19_NB extends Model CARMc19_N with all seven blood test results and AKI score (see appendix for equations and escalation policy Figure S5). We found that CARMc19 scores has good performance and our findings tentatively suggest that a NEWS2 threshold of 5+ appears to strike a reasonable balance between sensitivity and specificity. Model CARMc19_NB was more sensitive with similar specificity than the CARMc19_N model.

CARMc19 scores performed better than our previous CARM models [6] [7] because of additional NEWS2 variables (oxygen flow rate & oxygen scale 2) and COVID-19 status. A recent systematic review identified models to predict mortality from COVID-19 with c-statistics that ranged from 0.87 to 1 [20]. However, despite these high c-statistics, the review authors cautioned against the use of these models in clinical practice because of the high risk of bias and poor reporting of studies which are likely to have led to optimistic results [20].

The main advantages of our models are that they are designed to incorporate data which are already available in the patient's electronic health record thus placing no additional data collection or computational burden on clinicians, and are readily automated. Nonetheless, we emphasise that our computer-aided risk scores are not designed to replace clinical judgement. They are intended and designed to support, not subvert, the clinical decision-making process and can be always overridden by clinical concern [5,21]. The working hypothesis for our models is that they may enhance situational awareness of mortality by processing information already available without impeding the workflow of clinical staff, especially as our approach offers a faster and less expensive assessment of in-hospital mortality risk than current laboratory tests which may be more practical to use for large numbers of people.

For peer review only

There are limitations in relation to our study. We identified COVID-19 based on ICD-10 code ‘U071’ which was determined by COVID-19 swab test results (hospital or community) and clinical judgment and so our findings are constrained by the accuracy of these methods [22,23]. This does, however, allow the algorithm to take account of the entry of diagnostic information by the clinician including radiology findings as input variables if the swab result is negative. We used the index NEWS2 data in our models, but vital signs and blood test results are repeatedly updated for each patient according to hospital protocols. Although we developed models using one hospital’s data and validated into another hospital’s data, the extent to which changes in vital signs over time reflect changes in mortality risk need to be incorporated in our models requires further study. Our two hospitals are part of the same NHS Trust and this may undermine the generalisability of our findings, which merit further external validation.

Although we focused on in-hospital mortality (because we aimed to aid clinical decision making in the hospital), the impact of this selection bias needs to be assessed by capturing out-of-hospital mortality by linking death certification data and hospital data. CARMc19, like other risk scores, can only be an aid to the decision-making process of clinical teams [11,24] and its usefulness in clinical practice remains to be seen.

The next phase of this work is to field test CARMc19 scores by carefully engineering it into routine clinical practice to see if it does enhance the quality of care for acutely ill patients, whilst noting any unintended consequences.

Conclusion

We have developed a validated CARMc19 score with good performance characteristics for predicting the risk of in-hospital mortality following an emergency medical admission using the patient’s first, electronically recorded, vital signs and blood tests results. Since the CARMc19 scores place no additional data collection burden on clinicians and is readily automated, it may now be carefully introduced and evaluated in hospitals with sufficient informatics infrastructure.

For peer review only

Competing Interests

The authors declare no conflicts of interest. All authors have completed the (available on request from the first author) and declare: no support from any organisation for the submitted work [other than the funders described below]; no financial relationships with any organisations that might have an interest in the submitted work in the previous three years; and no other relationships or activities that could appear to have influenced the submitted work.

Funding

This research was supported by the Health Foundation. The Health Foundation is an independent charity working to improve the quality of healthcare in the UK. This research was also supported by the National Institute for Health Research (NIHR) Yorkshire and Humber Patient Safety Translational Research Centre (NIHR Yorkshire and Humber PSTRC). The views expressed in this article are those of the author(s) and not necessarily those of the NHS, the Health Foundation, the NIHR, or the Department of Health.

Role of the Funding Source

The funders of the study had no role in study design, data collection, data analysis, data interpretation, or writing of the report.

Author Contributions

DR and MAM had the original idea for the work. KB, RH provided the data extracts. MF undertook the statistical analyses with support from MAM. MF, MAM, and DR wrote the first draft of the paper. DR provided clinical perspectives. All others contributed to the final paper and have approved the final version. DR & MF will act as study guarantors.

Transparency Declaration

The lead author (the manuscript's guarantor) affirms that the manuscript is an honest, accurate, and transparent account of the study being reported and that no important aspects of the study have been omitted.

Ethical Approval

This study was deemed to be exempt from ethical approval because it was classified as an evaluation. Furthermore, this study used already de-identified data from an ongoing study involving NEWS which received ethical approval from Health Research Authority (HRA) and Health and Care Research Wales (HCRW) (reference number 19/HRA/0548).

Patient Involvement: None.

Data Sharing: Our data sharing agreement is with York hospital and does not permit us to share the data used in this paper.

For peer review only

Figure 1 Receiver Operating Characteristic curve for CARMc19_N and CARMc19_NB in predicting the risk of mortality in the development dataset

Note: predicted probability at NEWS2 thresholds 4+ [0.09], 5+ [0.11], 6+ [0.14] (sensitivity, specificity)

Figure 2 External validation of CARMc19_N and CARMc19_NB models, respectively for predicting the risk of mortality

NB: We limit the risk of mortality to 0.30 for visualisation purpose because beyond this point, we have few patients.

References

1 Gorbalenya AE, Baker SC, Baric RS, *et al.* The species Severe acute respiratory syndrome-related coronavirus: classifying 2019-nCoV and naming it SARS-CoV-2. Nat. Microbiol. 2020;**5**:536–44. doi:10.1038/s41564-020-0695-z

2 Onder G, Rezza G, Brusaferro S. Case-Fatality Rate and Characteristics of Patients Dying in Relation to COVID-19 in Italy. JAMA - J. Am. Med. Assoc. 2020;**323**:1775–6. doi:10.1001/jama.2020.4683

3 Vincent JL, Taccone FS. Understanding pathways to death in patients with COVID-19. Lancet Respir. Med. 2020;**8**:430–2. doi:10.1016/S2213-2600(20)30165-X

4 NHS. Royal College of Physicians: NHS England approves use of National Early Warning Score (NEWS) 2 to improve detection of acutely ill patients. 2017. https://www.rcplondon.ac.uk/news/nhs-england-approves-use-national-early-warning-score-news-2-improve-detection-acutely-ill

5 Royal College of Physicians. National Early Warning Score (NEWS): Standardising the assessment of acuteillness severity in the NHS - Report of a working party. 2012.

6 Faisal M, Richardson D, Scally A, *et al.* Performance of externally validated enhanced computer-aided versions of the National Early Warning Score in predicting mortality following an emergency admission to hospital in England: A cross-sectional study. *BMJ Open* 2019;**9**. doi:10.1136/bmjopen-2019-031596

7 Faisal M, Scally A, Jackson N, *et al.* Development and validation of a novel computer-aided score to predict the risk of in-hospital mortality for acutely ill medical admissions in two acute hospitals using their first electronically recorded blood test results and vital signs: a cross-section. *BMJ Open (accepted Oct 2018)* 2018.

8 Faisal M, Khatoon B, Scally A, *et al.* A prospective study of consecutive emergency medical admissions to compare a novel automated computer-aided mortality risk score and clinical judgement of patient mortality risk. *BMJ Open* 2019;**9**:e027741. doi:10.1136/bmjopen-2018-027741

9 StatCorp. Stata: Release 14. Statistical Software. College Station, TX: StataCorp LP. 2016.

10 Steyerberg EW. *Clinical Prediction Models. A practical approach to development, validation and updating.* Springer 2008.

11 Hanley JA, McNeil BJ. The meaning and use of the area under a receiver operating characteristic (ROC) curve. *Radiology* 1982;**143**:29–36. doi:10.1148/radiology.143.1.7063747

12 Robin X, Turck N, Hainard A, *et al.* pROC: an open-source package for R and S+ to analyze and compare ROC curves. *BMC Bioinformatics* 2011;**12**:77. doi:10.1186/1471-2105-12-77

13 R Development Core Team. R: A language and environment for statistical computing. R

For peer review only

Foundation for Statistical Computing http://www.r-project.org/. 2015.

14 Steyerberg EW, Harrell FE, Borsboom GJJ., *et al.* Internal validation of predictive models: Efficiency of some procedures for logistic regression analysis. *J Clin Epidemiol* 2001;**54**:774–81. doi:10.1016/S0895-4356(01)00341-9

15 Harrell FE. rms: Regression Modeling Strategies http://cran.r-project.org/package=rms. 2015.

16 National Institute for Health and Care Excellence. Sepsis Quality Standard [QS161]. Published September 2017. https://www.nice.org.uk/guidance/qs161/resources/sepsis-pdf-75545595402181

17 NHS England. Sepsis Guidance implementation advice for adults: NHS England, Sept 17. https://www.england.nhs.uk/wp-content/uploads/2017/09/sepsis-guidance-implementation-advice-for-adults.pdf

18 Sing T, Sander O, Beerenwinkel N, *et al.* ROCR: visualizing classifier performance in R. *Bioinformatics* 2005;**21**:3940–1. doi:10.1093/bioinformatics/bti623

19 Moons KGM, Altman DG, Reitsma JB, *et al.* Transparent Reporting of a multivariable prediction model for Individual Prognosis Or Diagnosis (TRIPOD): Explanation and Elaboration. *Ann Intern Med* 2015;**162**:W1. doi:10.7326/M14-0698

20 Wynants L, Van Calster B, Collins GS, *et al.* Prediction models for diagnosis and prognosis of covid-19: Systematic review and critical appraisal. *BMJ* 2020;**369**:18. doi:10.1136/bmj.m1328

21 Balamuth F, Alpern ER, Abbadessa MK, *et al.* Improving Recognition of Pediatric Severe Sepsis in the Emergency Department: Contributions of a Vital Sign-Based Electronic Alert and Bedside Clinician Identification. *Ann Emerg Med* 2017;**70**:759-768.e2. doi:10.1016/j.annemergmed.2017.03.019

22 Corfield AR, Lees F, Zealley I, *et al.* Utility of a single early warning score in patients with sepsis in the emergency department. 2012.

23 Churpek MM, Snyder A, Han X, *et al.* Quick Sepsis-related Organ Failure Assessment, Systemic Inflammatory Response Syndrome, and Early Warning Scores for Detecting Clinical Deterioration in Infected Patients outside the Intensive Care Unit. *Am J Respir Crit Care Med* 2017;**195**:906–11. doi:10.1164/rccm.201604-0854OC

24 Brabrand M, Folkestad L, Clausen NG, *et al.* Risk scoring systems for adults admitted to the emergency department: a systematic review. *Scand J Trauma Resusc Emerg Med* 2010;**18**:8. doi:10.1186/1757-7241-18-8

For peer review only

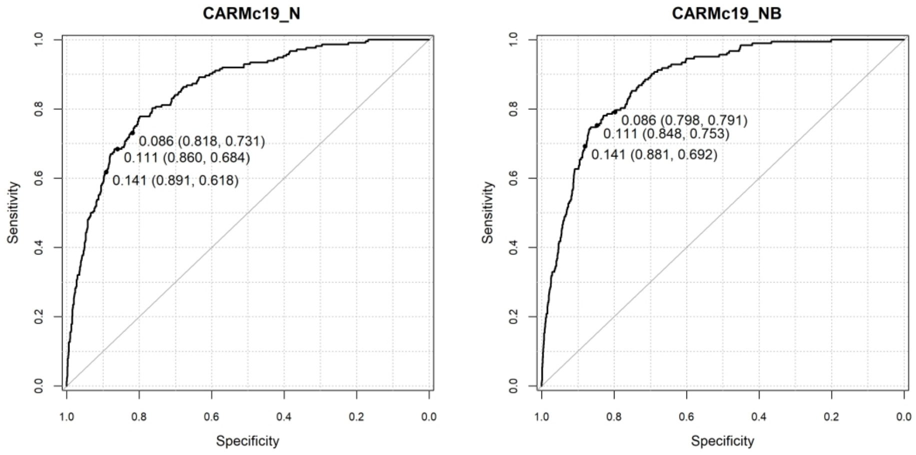

Figure 1 Receiver Operating Characteristic curve for CARMc19_N and CARMc19_NB in predicting the risk of mortality in the development dataset
Note: predicted probability at NEWS2 thresholds 4+ [0.09], 5+ [0.11], 6+ [0.14] (sensitivity, specificity)

304x152mm (300 x 300 DPI)

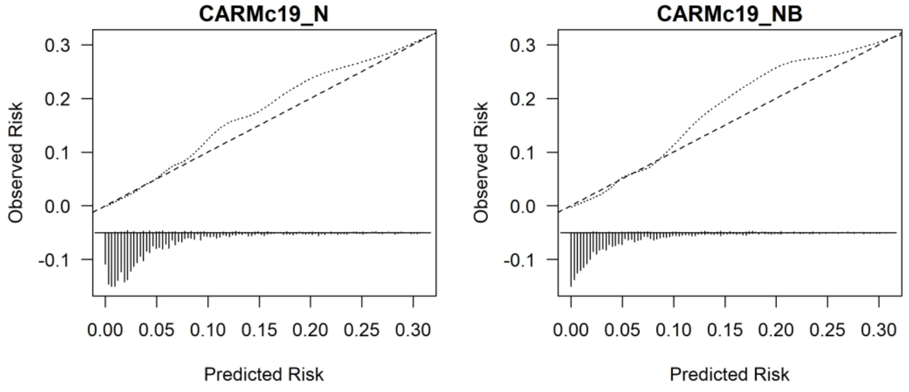

Figure 2 External validation of CARMc19_N and CARMc19_NB models, respectively for predicting the risk of mortality
NB: We limit the risk of mortality to 0.30 for visualisation purpose because beyond this point, we have few patients.

228x101mm (300 x 300 DPI)

Supplementary Material

Model CARMc19_N: predicting in-hospital mortality using vital signs only (N)

$$Logit(Died) = 10.671 + 9.262 * Male + 0.044 * Age + 0.069 * NEWS2 + 0.845 * log(Respiratory\ Rate) - 0.276 * Temperature - 0.022 * log(Diastolic\ pressure) - 1.524 * log(Systolic\ pressure) + 0.438 * log(Pulse\ rate) - 0.044 * Oxygen\ Saturations + 0.125 * Oxygen\ Supplementation + 0.487 * Baseline\ Confusion - 19.329 * New\ Confusion + 0.789 * Pain + 0.476 * Voice + 1.608 * Unconscious + 0.349 * Oxygen\ Scale\ 2 + 0.056 * Oxygen\ Flow\ Rate - 8.947 * COVID19 - 0.096 * Male * Oxygen\ Saturation + 3.856 * log(Systolic\ pressure) * New\ Confusion + 0.085 * Oxygen\ Saturation * COVID19 + 0.036 * Age * COVID19$$

Model CARMc19_NB: predicting in-hospital mortality using vital signs (N) and blood test results (B)

$$Logit(Died) = 14.241 - 1.478 * Male + 0.041 * Age - 0.063 * Albumin + 5.900 * log(Creatinine) - 0.002 * Haemoglobin - 0.830 * log(Potassium) - 0.043 * Sodium + 0.342 * log(White\ Cell\ Count) + 0.181 * log(Urea) + 0.434 * AKI\ Score\ 1 + 0.465 * AKI\ Score\ 2 + 0.406 * AKI\ Score\ 3 + 0.050 * NEWS2 + 0.729 * log(Respiratory\ Rate) - 0.257 * Temperature + 0.863 * log(Diastolic\ pressure) - 0.875 * log(Systolic\ pressure) + 0.066 * log(Pulse\ rate) - 0.055 * Oxygen\ saturation + 0.175 * Oxygen\ supplementation + 0.393 * Baseline\ Confusion - 0.414 * New\ Confusion + 1.085 * Pain + 1.024 * Voice + 2.635 * Unconscious + 0.611 * Oxygen\ Scale\ 2 + 0.026 * Oxygen\ Flow\ rate + 1.934 * COVID19 + 0.648 * Male * log(Urea) + 0.127 * New\ Confusion * log(White\ Cell\ Count)$$

We accounted for baseline difference in risk of mortality in the external validation data by subtracting (CARMc19_N: 0.24 and CARMc19_NB:0.27) from the CARMc19 logit models using an iterative procedure described elsewhere[1].

1. Faisal M, Howes R, Steyerberg EW, Richardson D, Mohammed MA. Using routine blood test results to predict the risk of death for emergency medical admissions to hospital: an external model validation study. QJM [Internet]. 2017 Jan 1 [cited 2017 Oct 2];110(1):27–31. Available from: https://academic.oup.com/qjmed/article-lookup/doi/10.1093/qjmed/hcw110

For peer review only

Table S1: NEWS2 scoring chart

Physiological Parameters	3	2	1	0	1	2	3
Respiration Rate	≤8		9 - 11	12 - 20		21 - 24	≥25
SpO2 Scale 1 (%)	≤91	92 - 93	94 - 95	≥96			
SpO2 Scale 2 (%)	≤83	84 - 85	86 - 87	88 - 92<br>≥93 on Air	93 – 94 on oxygen	95 – 96 on oxygen	≥97 on oxygen
Oxygen Saturations	≤91	92 - 93	94 - 95	≥96			
Air or Oxygen?		Oxygen		Air			
Temperature	≤35.0		35.1 - 36.0	36.1 - 38.0	38.1 - 39.0	≥39.1	
Systolic BP	≤90	91 - 100	101 - 110	111 - 219			≥220
Heart Rate	≤40		41 - 50	51-90	91 - 110	111 - 130	≥131
Level of Consciousness				Alert			Voice, Pain, Confusion, or Unconscious

The NEWS [https://www.rcplondon.ac.uk/projects/outputs/national-early-warning-score-news] is based on a scoring system in which a score is allocated to the physiological measurements of vital signs already undertaken when patients present to or are being monitored in hospital. A score is allocated to each as they are measured, the magnitude of the score reflecting how extreme the parameter varies from the norm. This score is then aggregated and uplifted for people requiring oxygen.

Characteristic	Development dataset (YH)	Validation dataset (SH)	All
	N (%)	N (%)	N (%)
**Total emergency medical discharges between 11 Mar 20 to 13 June 20**	3952	2528	6480
**Excluded: No NEWS2 recorded (%)**	13 (0.3)	6 (0.2)	19 (0.3)
**Excluded: First NEWS2 after 24 hours of admission (%)**	15 (0.4)	2 (0.1)	17 (0.3)
**Excluded: First blood test results after 4 days of admission (%)**	786 (20.0)	403 (15.9)	1189 (18.3)
**Total excluded (%)**	814 (20.6)	411 (16.3)	1225 (18.9)
**Total included (%)**	3138 (79.4)	2117 (84.7)	5255 (81.1)

Table S2 Number of emergency medical admissions included/excluded

For peer review only

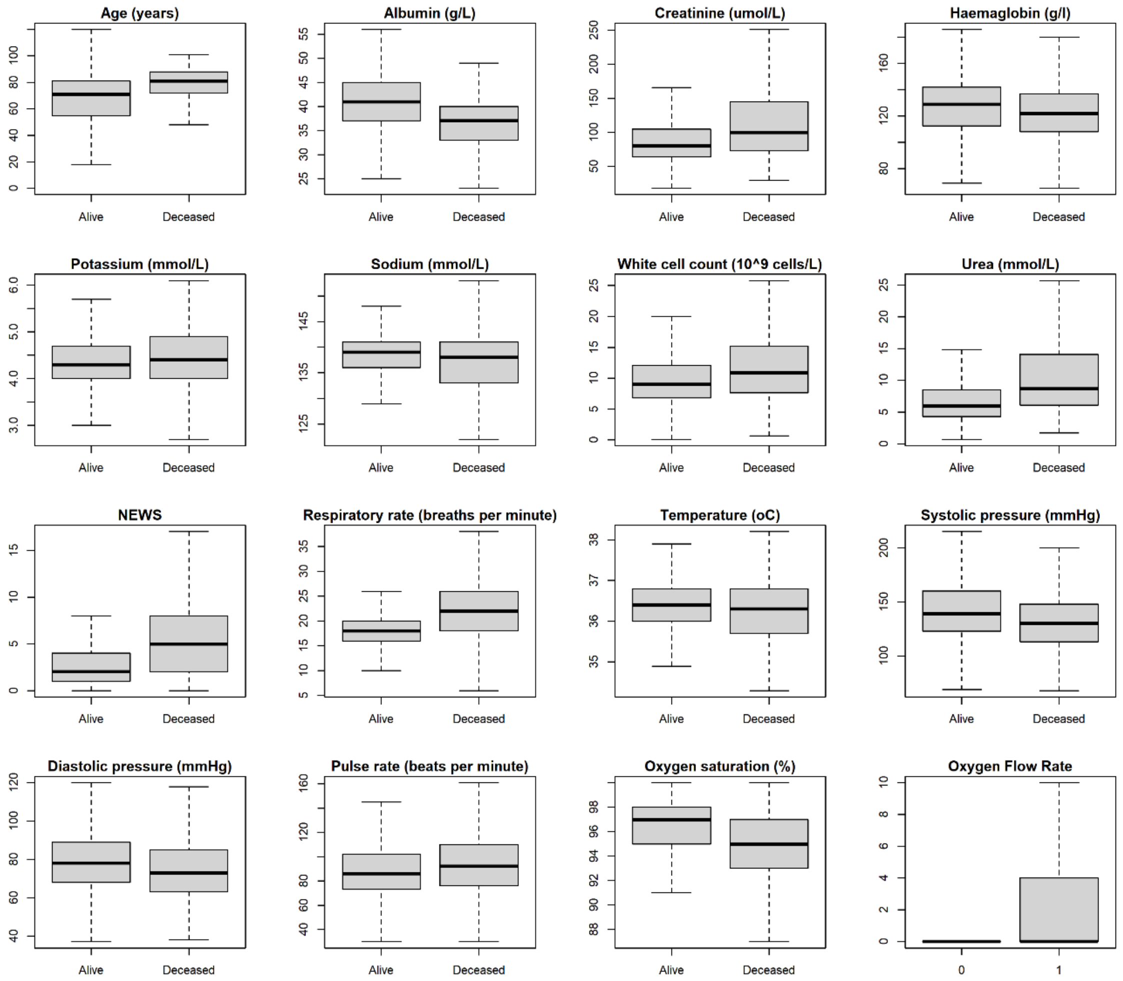

Figure S1 Boxplot for continuous covariates without outliers with respect to discharge status (alive/deceased) for development dataset.

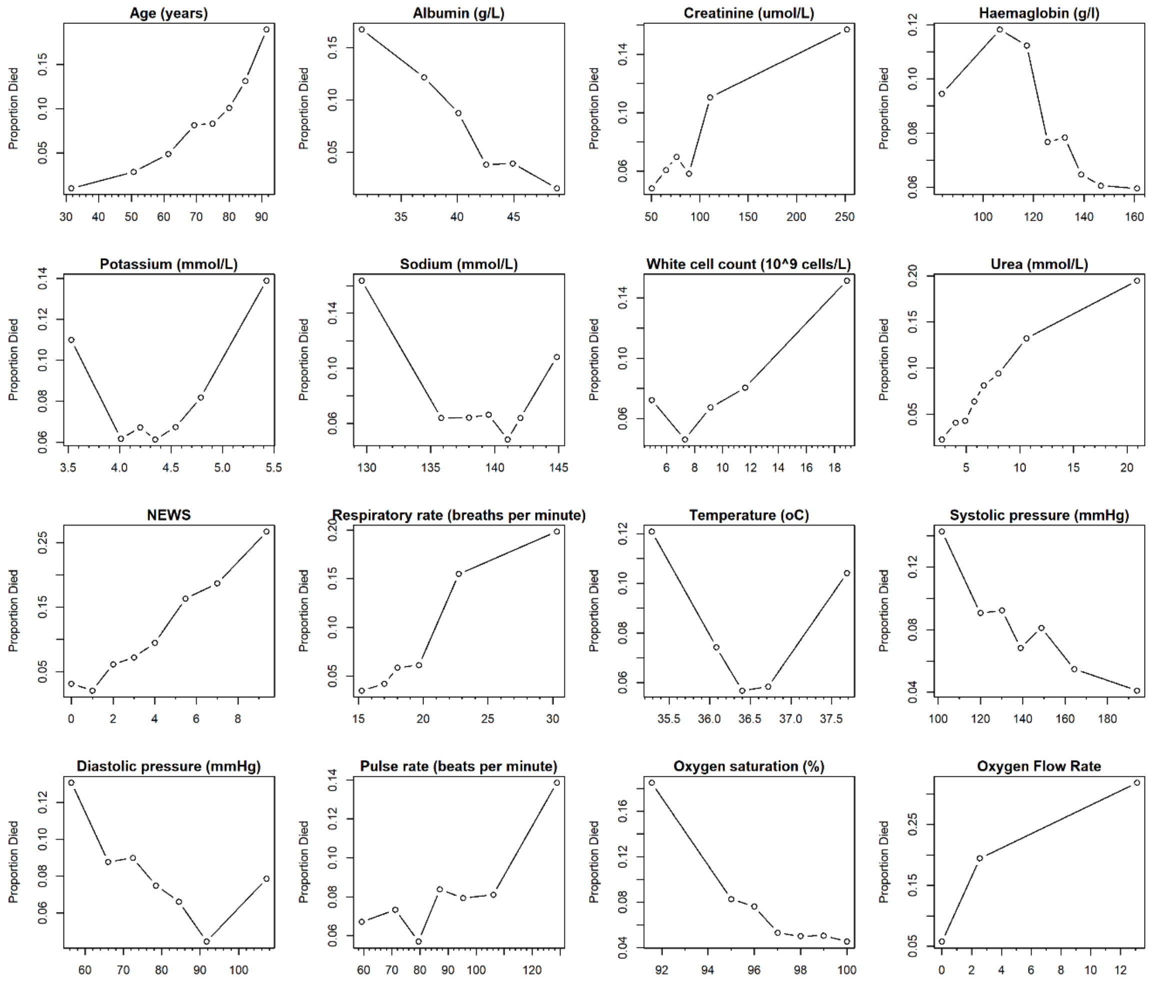

Figure S2 Scatter plots showing the observed risk of in-hospital mortality with continuous covariates for development dataset.

NB: y-axis range changes in each plot.

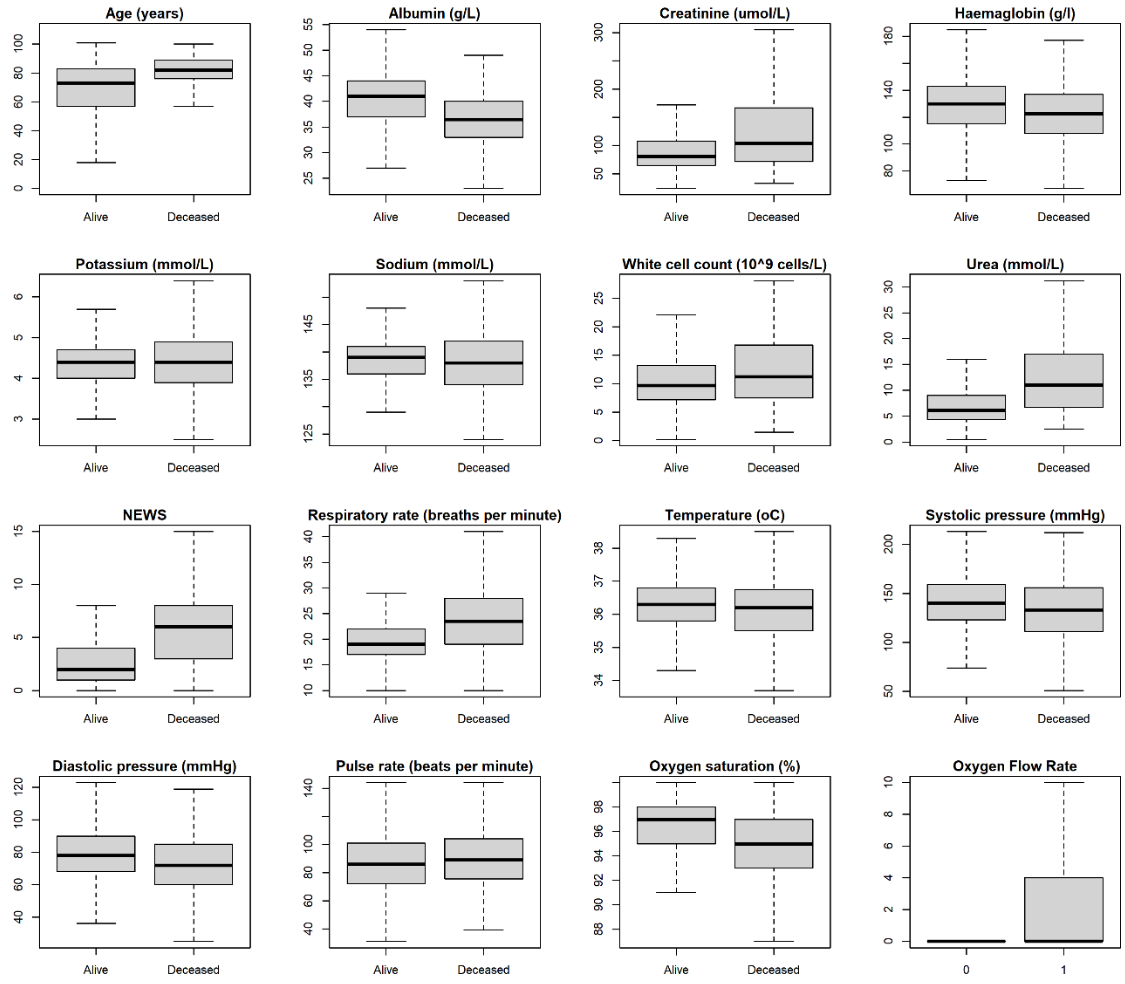

Figure S3 Boxplot for continuous covariates without outliers with respect to discharge status (alive/deceased) for validation dataset.

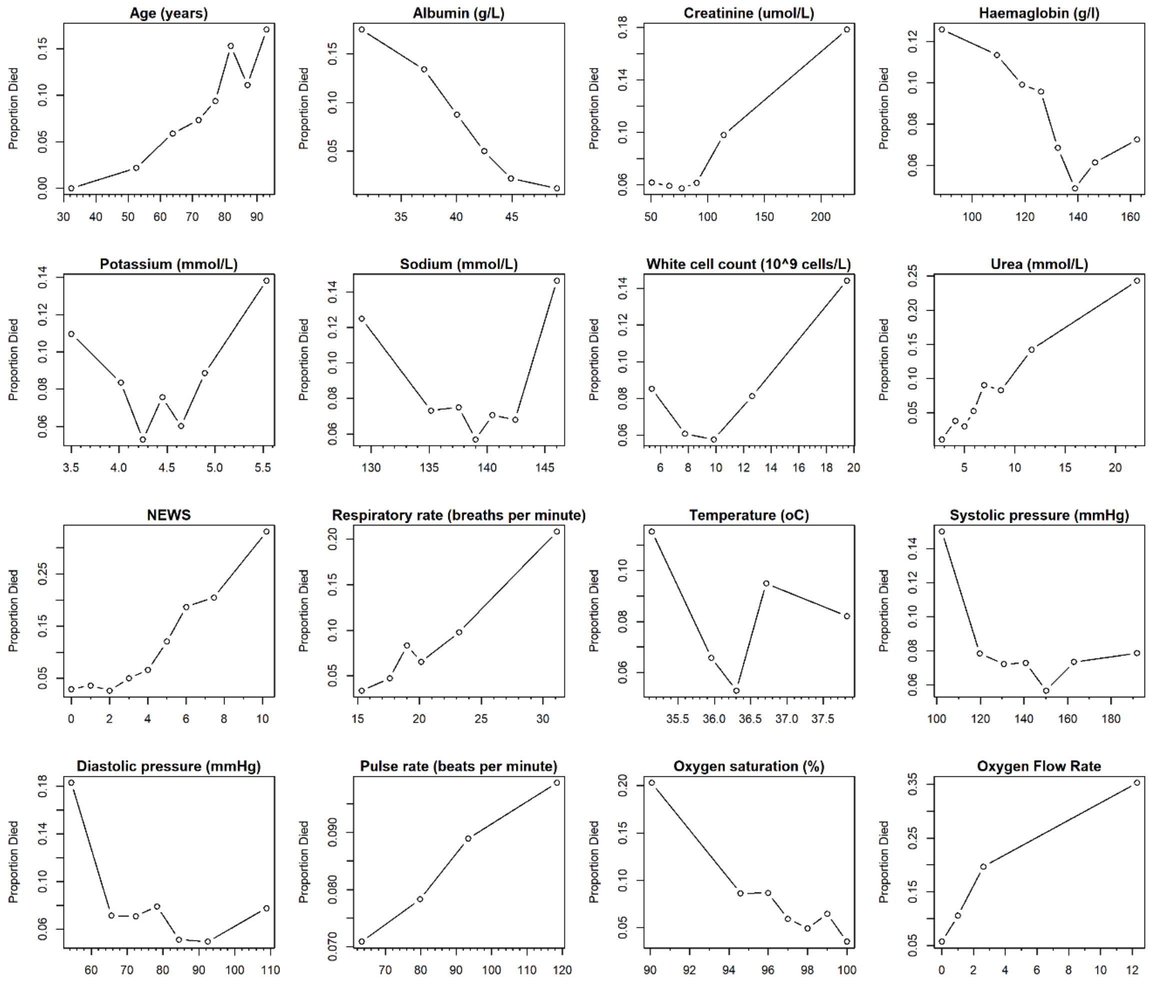

Figure S4 Scatter plots showing the observed risk of in-hospital mortality with continuous covariates for validation dataset.

NB: y-axis range changes in each plot.

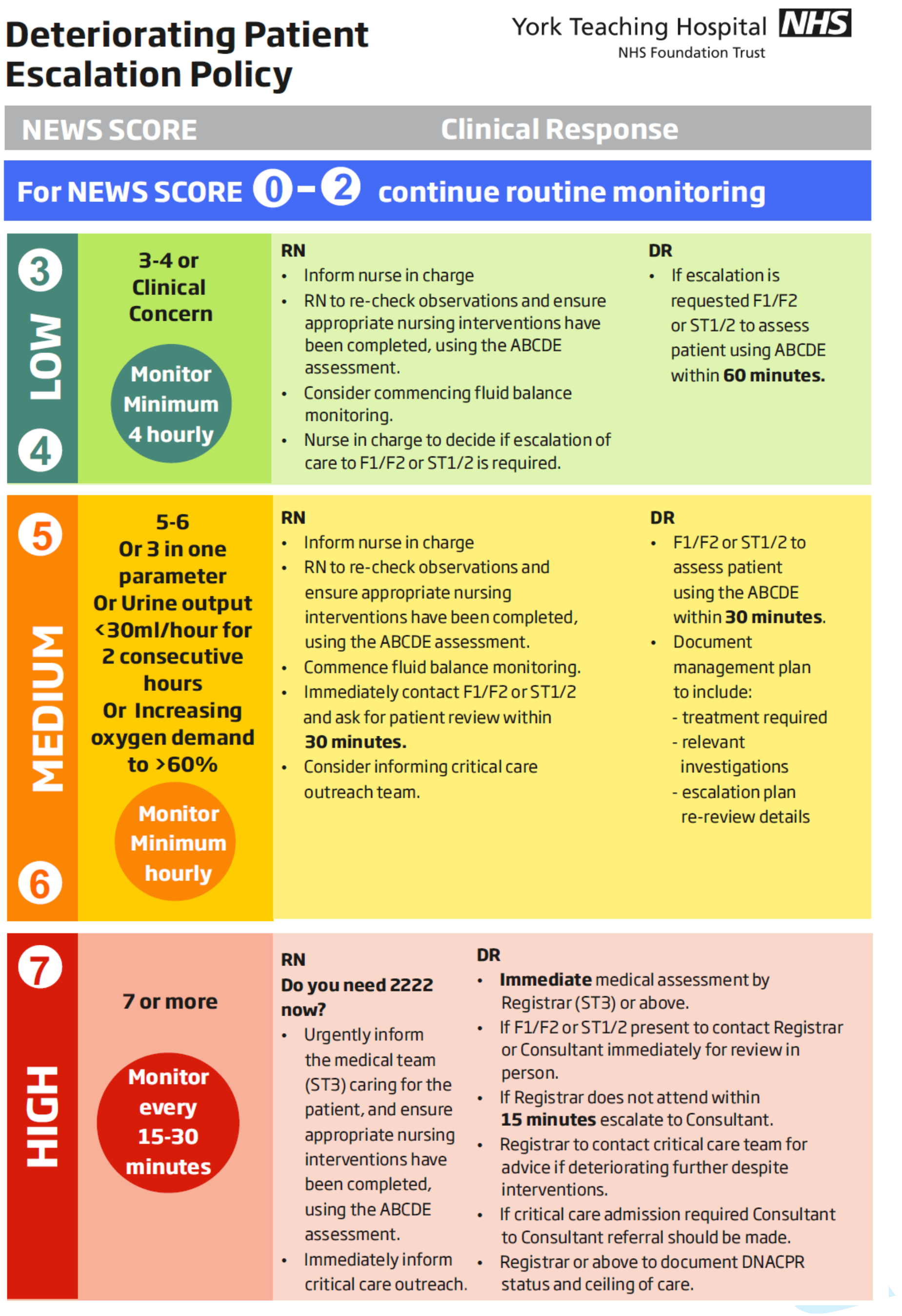

Figure S5 Escalation policy of deteriorating patients in York Teaching Hospital NHS Foundation Trust.

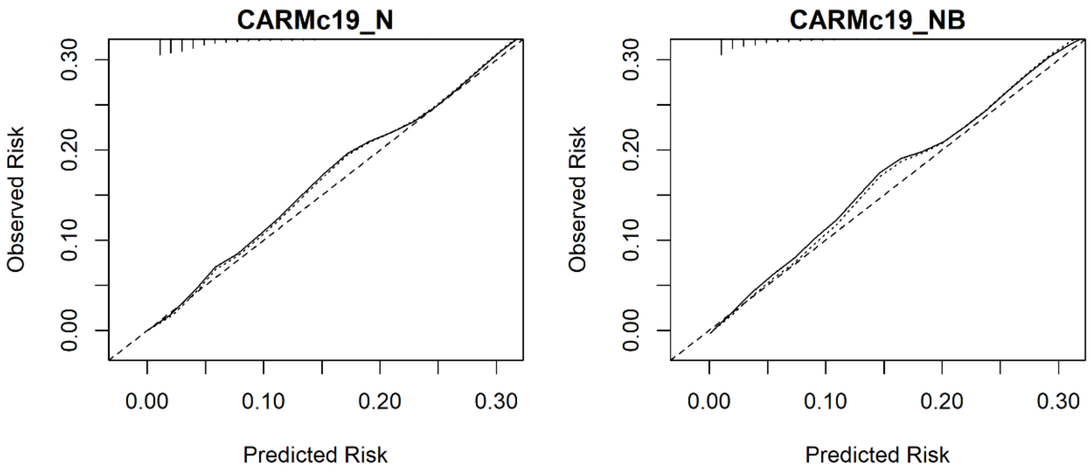

Figure S6 Calibration of CARMc19_N and CARMc19_NB models respectively for predicting the risk of mortality.

NB: We limit the risk of mortality to 0.30 for visualisation purpose because beyond this point, we have few patients.

For peer review only

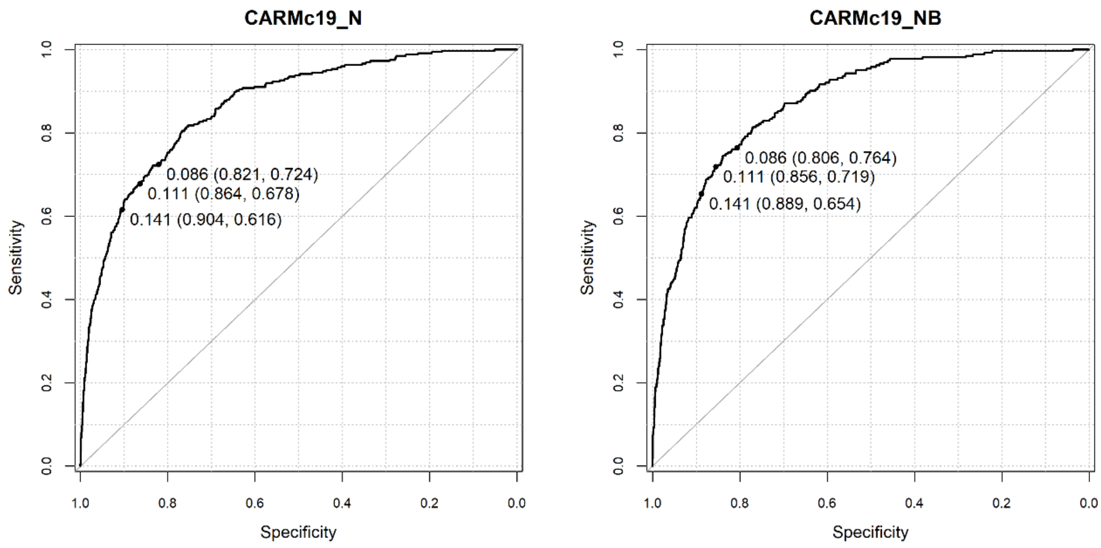

Figure S7 Receiver Operating Characteristic curve for CARMc19_N and CARMc19_NB in predicting the risk of mortality in the development dataset.

Note: predicted probability at NEWS2 thresholds 4+ [0.09], 5+[0.11], 6+[0.14] (sensitivity, specificity)

For peer review only

Model	Mean risk discharged alive	Mean risk discharged deceased	ARD	Scaled brier score	AUC (95% CIs)	Optimism-corrected c-statistic
CARMc19_N	0.06	0.31	0.25	0.24	0.86 (0.84 to 0.87)	0.85
CARMc19_NB	0.06	0.31	0.25	0.24	0.87 (0.85 to 0.89)	0.86

Table S3: Performance of CARMc19_N and CARMc19_NB models for predicting the risk of mortality in the development dataset.

ARD: Absolute risk difference; AUC: Area under the curve; CIs: Confidence intervals

Model	At NEWS score [predicted risk of death]	Number of deaths identified by the model	Sensitivity%	Specificity%	PPV	NPV	LR+	LR-
CARMc19_N	4+ [0.09]	880	72.4 (67.2 to 77.2)	82.1 (80.8 to 83.3)	26.6 (23.7 to 29.6)	97.1 (96.4 to 97.6)	4 (3.7 to 4.4)	0.3 (0.3 to 0.4)
CARMc19_N	5+ [0.11]	710	67.8 (62.4 to 72.9)	86.4 (85.2 to 87.5)	30.8 (27.5 to 34.4)	96.8 (96.1 to 97.3)	5 (4.4 to 5.6)	0.4 (0.3 to 0.4)
CARMc19_N	6+ [0.14]	544	61.6 (56.1 to 66.9)	90.4 (89.4 to 91.4)	36.6 (32.5 to 40.8)	96.3 (95.6 to 96.9)	6.4 (5.6 to 7.3)	0.4 (0.4 to 0.5)
CARMc19_NB	4+ [0.09]	750	76.4 (70.8 to 81.4)	80.6 (79.1 to 82)	26.4 (23.3 to 29.7)	97.4 (96.7 to 98)	3.9 (3.6 to 4.4)	0.3 (0.2 to 0.4)
CARMc19_NB	5+ [0.11]	602	71.9 (66 to 77.2)	85.6 (84.2 to 86.8)	31.2 (27.5 to 35)	97.1 (96.4 to 97.7)	5 (4.4 to 5.6)	0.3 (0.3 to 0.4)
CARMc19_NB	6+ [0.14]	496	65.4 (59.3 to 71.1)	88.9 (87.6 to 90)	34.8 (30.6 to 39.2)	96.6 (95.8 to 97.2)	5.9 (5.1 to 6.7)	0.4 (0.3 to 0.5)

Table S4 Sensitivity analysis of CARMc19_N and CARMc19_NB models for predicting the risk of mortality at NEWS2 thresholds (4,5,6) for development dataset.

PPV = Positive Predictive Value; NPV = Negative Predictive Value; LR+ = Positive Likelihood Ratio; LR- = Negative Likelihood Ratio

For peer review only

TRIPOD Checklist: Prediction Model Development and Validation

Section/Topic	Item		Checklist Item	Page
**Title and abstract**				
Title	1	D;V	Identify the study as developing and/or validating a multivariable prediction model, the target population, and the outcome to be predicted.	1
Abstract	2	D;V	Provide a summary of objectives, study design, setting, participants, sample size, predictors, outcome, statistical analysis, results, and conclusions.	3
**Introduction**				
Background and objectives	3a	D;V	Explain the medical context (including whether diagnostic or prognostic) and rationale for developing or validating the multivariable prediction model, including references to existing models.	5
	3b	D;V	Specify the objectives, including whether the study describes the development or validation of the model or both.	6
**Methods**				
Source of data	4a	D;V	Describe the study design or source of data (e.g., randomized trial, cohort, or registry data), separately for the development and validation data sets, if applicable.	6
	4b	D;V	Specify the key study dates, including start of accrual; end of accrual; and, if applicable, end of follow-up.	7
Participants	5a	D;V	Specify key elements of the study setting (e.g., primary care, secondary care, general population) including number and location of centres.	7
	5b	D;V	Describe eligibility criteria for participants.	7
	5c	D;V	Give details of treatments received, if relevant.	
Outcome	6a	D;V	Clearly define the outcome that is predicted by the prediction model, including how and when assessed.	8
	6b	D;V	Report any actions to blind assessment of the outcome to be predicted.	
Predictors	7a	D;V	Clearly define all predictors used in developing or validating the multivariable prediction model, including how and when they were measured.	7
	7b	D;V	Report any actions to blind assessment of predictors for the outcome and other predictors.	
Sample size	8	D;V	Explain how the study size was arrived at.	7
Missing data	9	D;V	Describe how missing data were handled (e.g., complete-case analysis, single imputation, multiple imputation) with details of any imputation method.	7
Statistical analysis methods	10a	D	Describe how predictors were handled in the analyses.	8
	10b	D	Specify type of model, all model-building procedures (including any predictor selection), and method for internal validation.	8
	10c	V	For validation, describe how the predictions were calculated.	8
	10d	D;V	Specify all measures used to assess model performance and, if relevant, to compare multiple models.	8,9
	10e	V	Describe any model updating (e.g., recalibration) arising from the validation, if done.	suppl
Risk groups	11	D;V	Provide details on how risk groups were created, if done.	
Development vs. validation	12	V	For validation, identify any differences from the development data in setting, eligibility criteria, outcome, and predictors.	8,9
**Results**				
Participants	13a	D;V	Describe the flow of participants through the study, including the number of participants with and without the outcome and, if applicable, a summary of the follow-up time. A diagram may be helpful.	10
	13b	D;V	Describe the characteristics of the participants (basic demographics, clinical features, available predictors), including the number of participants with missing data for predictors and outcome.	10,11
	13c	V	For validation, show a comparison with the development data of the distribution of important variables (demographics, predictors and outcome).	11
Model development	14a	D	Specify the number of participants and outcome events in each analysis.	10
	14b	D	If done, report the unadjusted association between each candidate predictor and outcome.	
Model specification	15a	D	Present the full prediction model to allow predictions for individuals (i.e., all regression coefficients, and model intercept or baseline survival at a given time point).	suppl
	15b	D	Explain how to the use the prediction model.	suppl
Model performance	16	D;V	Report performance measures (with CIs) for the prediction model.	11
Model-updating	17	V	If done, report the results from any model updating (i.e., model specification, model performance).	
**Discussion**				
Limitations	18	D;V	Discuss any limitations of the study (such as nonrepresentative sample, few events per predictor, missing data).	12
Interpretation	19a	V	For validation, discuss the results with reference to performance in the development data, and any other validation data.	12
	19b	D;V	Give an overall interpretation of the results, considering objectives, limitations, results from similar studies, and other relevant evidence.	12,13
Implications	20	D;V	Discuss the potential clinical use of the model and implications for future research.	13
**Other information**				
Supplementary information	21	D;V	Provide information about the availability of supplementary resources, such as study protocol, Web calculator, and data sets.	suppl
Funding	22	D;V	Give the source of funding and the role of the funders for the present study.	14

*Items relevant only to the development of a prediction model are denoted by D, items relating solely to a validation of a prediction model are denoted by V, and items relating to both are denoted D;V. We recommend using the TRIPOD Checklist in conjunction with the TRIPOD Explanation and Elaboration document.

For peer review only - http://bmjopen.bmj.com/site/about/guidelines.xhtml

BMJ Open

Development and validation of automated computer aided-risk scores to predict in-hospital mortality for emergency medical admisisons with COVID-19: a retrospective cohort development and validation study

Journal:	*BMJ Open*
Manuscript ID	bmjopen-2021-050274.R1
Article Type:	Original research
Date Submitted by the Author:	09-Oct-2021
Complete List of Authors:	Faisal, Muhammad; University of Bradford Faculty of Health Studies; Bradford Institute for Health Research<br>Mohammed, Mohammed ; University of Bradford Faculty of Health Studies; NHS Midlands and Lancashire Commissioning Support Unit<br>Richardson, Donald; York Teaching Hospital NHS Foundation Trust<br>Fiori, Massimo ; York Teaching Hospital NHS Foundation Trust<br>Beatson, Kevin; York Teaching Hospital NHS Foundation Trust
**Primary Subject Heading**:	Health informatics
Secondary Subject Heading:	Health informatics, Health services research, Intensive care, Research methods
Keywords:	COVID-19, Infection control < INFECTIOUS DISEASES, Health & safety < HEALTH SERVICES ADMINISTRATION & MANAGEMENT, Quality in health care < HEALTH SERVICES ADMINISTRATION & MANAGEMENT

SCHOLARONE™
Manuscripts

For peer review only - http://bmjopen.bmj.com/site/about/guidelines.xhtml

I, the Submitting Author has the right to grant and does grant on behalf of all authors of the Work (as defined in the below author licence), an exclusive licence and/or a non-exclusive licence for contributions from authors who are: i) UK Crown employees; ii) where BMJ has agreed a CC-BY licence shall apply, and/or iii) in accordance with the terms applicable for US Federal Government officers or employees acting as part of their official duties; on a worldwide, perpetual, irrevocable, royalty-free basis to BMJ Publishing Group Ltd ("BMJ") its licensees and where the relevant Journal is co-owned by BMJ to the co-owners of the Journal, to publish the Work in this journal and any other BMJ products and to exploit all rights, as set out in our licence.

The Submitting Author accepts and understands that any supply made under these terms is made by BMJ to the Submitting Author unless you are acting as an employee on behalf of your employer or a postgraduate student of an affiliated institution which is paying any applicable article publishing charge ("APC") for Open Access articles. Where the Submitting Author wishes to make the Work available on an Open Access basis (and intends to pay the relevant APC), the terms of reuse of such Open Access shall be governed by a Creative Commons licence – details of these licences and which Creative Commons licence will apply to this Work are set out in our licence referred to above.

Other than as permitted in any relevant BMJ Author's Self Archiving Policies, I confirm this Work has not been accepted for publication elsewhere, is not being considered for publication elsewhere and does not duplicate material already published. I confirm all authors consent to publication of this Work and authorise the granting of this licence.

For peer review only

Development and validation of automated computer aided-risk scores to predict in-hospital mortality for emergency medical admisisons with COVID-19: a retrospective cohort development and validation study

Authors

Muhammad Faisal, PhD
Senior Research Fellow in Biostatistics
Faculty of Health Studies, University of Bradford, Bradford, UK
Bradford Institute for Health Research Bradford, UK
NIHR Yorkshire and Humber Patient Safety Translational Research Centre (YHPSTRC), Bradford, UK
Wolfson Centre for Applied Health Research, Bradford, UK
E-mail: M.Faisal1@bradford.ac.uk

Mohammed A Mohammed, PhD
Professor of Healthcare Quality & Effectiveness
Faculty of Health Studies, University of Bradford, Bradford, UK
NHS Midlands and Lancashire Commissioning Support Unit,
The Strategy Unit,
Kingston House,
West Bromwich, B70 9LD, UK
E-mail: M.A.Mohammed5@Bradford.ac.uk

Donald Richardson, FRCP
Deputy Medical Director , Chief Clinical Information Officer, Consultant Renal Physician
Department of Renal Medicine, York Teaching Hospitals NHS Foundation Trust
E-mail: drichardson@doctors.org.uk

Massimo Fiori
Senior Analyst/ Programmer
York Teaching Hospitals NHS Foundation Trust, England UK
E-mail: massimo.fiori@york.nhs.uk

Kevin Beatson, MSc
Development Manager
York Teaching Hospitals NHS Foundation Trust, England UK
E-mail: Kevin.Beatson@York.NHS.uk

Correspondence to: M A Mohammed

For peer review only

Abstract

Objectives: There are no established mortality risk equations specifically for unplanned emergency medical admissions which include patients with the novel coronavirus SARS-19(COVID-19). We aim to develop and validate a computer-aided risk score (CARMc19) for predicting mortality risk by combining COVID-19 status, the first electronically recorded blood test results and the National Early Warning Score (NEWS2).

Design: Logistic regression model development and validation study

Setting: Two acute hospitals (YH – model development data; SH – external validation data).

Participants: Adult (>=16 years) medical admissions discharged over a 24 month period with electronic NEWS and blood test results recorded on admission. We used logistic regression modelling to predict the risk of in-hospital mortality using two models: 1) CARMc19_N: age + sex + NEWS2 including subcomponents + COVID19; 2) CARMc19_NB: CARMc19_N in conjunction with seven blood test results and acute kidney injury score. Model performance was evaluated according to discrimination (c-statistic), calibration (graphically), and clinical usefulness at NEWS2 thresholds of 4+, 5+, 6+.

Results: The risk of in-hospital mortality following emergency medical admission was similar in development and validation datasets (8.4% vs 8.2%). The c-statistics for predicting mortality for CARMc19_NB is better than CARMc19_N in the validation dataset (CARMc19_NB=0.88(95%CI 0.86-0.90) vs CARMc19_N=0.86(95%CI 0.83-0.88)). Both models had good calibration (CARMc19_NB=1.01(95%CI 0.88-1.14) & CARMc19_N:0.95(95%CI 0.83-1.06)). At all NEWS2 thresholds (4+, 5+, 6+) model CARMc19_NB had better sensitivity and similar specificity.

Conclusions: We have developed a validated CARMc19 scores with good performance characteristics for predicting the risk of in-hospital mortality. Since the CARMc19 scores place no additional data collection burden on clinicians, it may now be carefully introduced and evaluated in hospitals with sufficient informatics infrastructure.

Keywords: vital signs, national early warning score, emergency admission, novel coronavirus SARS-19, computer-aided national early warning score

Article Summary

- This study provides a computer-aided risk of in-hospital mortality for unplanned admissions with the novel coronavirus SARS-19(COVID-19) using NEWS2 and routine blood test results.
- About 20-30% of admissions do not have both NEWS2 and blood test results and so we have developed two scores (CARMc19_N and CARMc19_NB) reflecting those with/without blood test results.
- COVID-19 patients was determined by COVID-19 swab test results (hospital or community) and clinical judgment and so our findings are constrained by the accuracy of these methods.
- Our two hospitals are part of the same NHS Trust and this may undermine the generalisability of our findings, which merit further external validation.
- CARMc19 scores place no additional data collection burden on clinicians and are readily automated.

For peer review only

Introduction

The novel coronavirus SARS-19 produced 'COVID-19' infection in individuals with symptoms that has challenged health care systems globally (Coronaviridae Study Group of the International Committee on Taxonomy of Viruses[1]). COVID-19 patients admitted to the hospital during the early stages of the pandemic were at severe risk of developing the severe disease with life-threatening respiratory and/or multi-organ failure [2,3] with a high risk of mortality.

Early diagnosis and management of COVID-19 patients was key in providing high-quality care, which included palliative care, isolation, and escalation to critical care. Early Warning Scores (EWS) are commonly used in hospitals worldwide [4], and in the National Health Service (NHS) hospitals in England, the patient's National Early Warning Score (NEWS) is used to identify patients at risk of deterioration [5]. We have developed two automated risk equations to predict the patient's risk of in-hospital mortality (CARM_N & CARM_NB) using NEWS only (CARM_N) [6] and NEWS + blood test results (CARM_NB) [7] following emergency medical admission to hospital. We found CARM_NB performed similar to consultant clinicians [8].

NEWS2 was published in December 2017 as an update to NEWS [4] that considered new confusion or delirium and allocated three points (the maximum for a single variable). NEWS2 also offers two scales for oxygen saturation (scale 1 and scale 2). Scale 2 is used for patients at risk of hypercapnic respiratory failure who have a lower oxygen saturation target of 88–92%.

Whilst hospitals continued to use NEWS2 during the COVID-19 pandemic, little was known at the time about how NEWS2 and CARM scores perform in monitoring COVID-19 patients. In this study, we aimed to develop and validate an automated computer aided risk score (CARMc19) using on admission NEWS2 and blood test results for predicting mortality in our patient cohort that included a large number with a diagnosis of COVID-19. This approach is clinically useful because it places no additional data collection burden on staff for monitoring COVID-19 patients. It must be stressed that this algorithm was developed at a time that predated wide spread vaccination and the development of other evidenced based treatments for COVID -19 disease. The PREVENT study was ongoing in the trust during the development of this algorithm [9].

For peer review only

Methods

Setting & data

Our cohorts of emergency medical admissions are from two acute hospitals which are approximately 65 kilometres apart in the Yorkshire & Humberside region of England – Scarborough hospital (n~300 beds) and York Hospital (YH) (n~700 beds), managed by York Teaching Hospitals NHS Foundation Trust. We selected these hospitals because they had electronic NEWS2, collected as part of the patient's process of care since April 2019, and were agreeable to the study.

We considered all consecutive adult (age≥18 years) non-elective or emergency medical admissions discharged over a course of three months (11 March 2020 to 13 June 2020) with electronic NEWS2. For each emergency admission, we obtained a pseudonymised patient identifier, patient's age (years), sex (male/female), discharge status (alive/dead), admission and discharge date and time, diagnoses codes based on the 10th revision of the International Statistical Classification of Diseases (ICD-10), NEWS2 (including its subcomponents respiratory rate, temperature, systolic pressure, pulse rate, oxygen saturation, oxygen supplementation, oxygen scales 1 & 2, and alertness including confusion), blood test results (albumin, creatinine, haemoglobin, potassium, sodium, urea, and white cell count), and Acute Kidney Injury (AKI) score.

The diastolic blood pressure was recorded at the same time as systolic blood pressure. Historically, diastolic blood pressure has always been a routinely collected physiological variable on vital sign charts and is still collected where electronic observations are in place. NEWS2 produces integer values that range from 0 (indicating the lowest severity of illness) to 20 (the maximum NEWS2 value possible) (see Table S1 in supplementary material). The index NEWS2 was defined as the first electronically recorded NEWS2 within ±24 hours of the admission time. We excluded records where the index NEWS2 (or blood test results) was not within ±24 hours (±96 hours) or was missing/not recorded at all (see Table S2). The ICD-10 code 'U071' was used to identify records with COVID-19. We searched primary and secondary ICD-10 codes for 'U071' for identifying COVID-19.

Statistical Modelling

We began with exploratory analyses including box plots and line plots to show the relationship between covariates and risk of in-hospital mortality. We developed two logistic regression models, known as CARMc19_N and CARMc19_NB, to predict the risk of in-hospital mortality with following covariates: 1) Model CARMc19_N uses age + sex + COVID-19 (yes/no) + NEWS2 including sub components; 2) Model CARMc19_NB extends Model CARMc19_N with all seven blood test results and AKI score. The primary rationale for using these variables is that they are routinely collected as part of

For peer review only - http://bmjopen.bmj.com/site/about/guidelines.xhtml

process of care and their inclusion in our statistical models is on clinical grounds as opposed to the statistical significance of any given covariate.

We used the *qladder* function (Stata [10]), which displays the quantiles of a transformed variable against the quantiles of a normal distribution according to the ladder powers $(x^3, x^2, x^1, x, \sqrt{x}, \log(x), x^{-1}, x^{-2}, x^{-3})$ for each continuous covariate and chose the following transformations: (creatinine)-1/2, $\log_e$(potassium), $\log_e$(white cell count), $\log_e$(urea), $\log_e$ (respiratory rate), $\log_e$(pulse rate), $\log_e$(systolic blood pressure), and $\log_e$(diastolic blood pressure). We used an automated approach to search for all two-way interactions and incorporated those interactions which were statistically significant ($p<0.001$) from the MASS library [12] in *R* [13].

We developed both models using York Hospital (YH) data (development dataset) and externally validated their performance on Scarborough Hospital (SH) data (validation dataset). The hospitals are part of the same NHS Trust but are geographically separated by about 65 kilometres (40 miles).

We report discrimination and calibration statistics as performance measures for these models [11].

Discrimination relates to how well a model can separate – or discriminate between – those who died and those who did not and is given by the area under the Receiver Operating Characteristics (ROC) curve (AUC) or c-statistic. The ROC curve is a plot of the sensitivity (true positive rate) versus 1-specificity (false positive rate) for consecutive predicted risks. A c-statistic of 0.5 is no better than tossing a coin, whilst a perfect model has a c-statistic of 1. In general, values less than 0.7 are considered to show poor discrimination, values of 0.7 to 0.8 can be described as reasonable, and values above 0.8 suggest good discrimination [12]. The 95% confidence interval for the c-statistic was derived using DeLong's method as implemented in the *pROC* library [13] in *R* [14].

Calibration measures a model's ability to generate predictions that are, on average, close to the average observed outcome and can be readily seen on a scatter plot (y-axis = observed risk, x-axis = predicted risk). Perfect predictions should be on the 45° line. We internally validated and assessed the calibration for all the models using the bootstrapping approach [15,16]. The overall statistical performance was assessed using the scaled Brier score which incorporates both discrimination and calibration [11]. The Brier score is the squared difference between actual outcomes and predicted risk of death, scaled by the maximum Brier score such that the scaled Brier score ranges from 0–100%. Higher values indicate superior models.

The recommended threshold for detecting deteriorating patients and sepsis is NEWS2≥5 [17,18]. Therefore, we assessed the sensitivity, specificity, positive and negative predictive values and likelihood ratios for these models at NEWS2 threshold of 4+,5+, and 6+ [19]. We followed the TRIPOD

For peer review only

guidelines for reporting of model development and validation [20]. We used Stata [10] for data cleaning and *R* [14] for statistical analysis.

Results

Cohort Characteristics

The number of non-elective discharges was 6444 over 3 months. For the development of CARMc19_N, we excluded 36 (0.6%) admissions because the index NEWS2 was not recorded within ±24 hours of the admission date/time, or these data were missing or not recorded at all (see Table S2). Likewise, for the development of CARMc19_NB, we further excluded 1189 (18.3%) of admissions because the first blood test results were not recorded within ±96 hours of the admission date/time, or they were missing or not recorded at all (see Table S2).

The characteristics of the admissions included in our study are shown in Table 1. Emergency admissions in the validation dataset were older than those in development dataset (69.6 years vs 67.4 years), less likely to be male (49.5% vs 51.2%), had higher index NEWS2 (3.2 vs 2.8), higher prevalence of COVID-19 (11.0% vs 8.7%) but similar in-hospital mortality (8.4% vs 8.2%). See accompanying scatter and boxplots in Figure S1 to S4 (supplemental digital content).

For peer review only

Characteristic	Development dataset (YH)	Validation dataset (SH)	Degree of freedom (df)	p-value
N	3924	2520		-
Male (%)	2010 (51.2)	1247 (49.5)	1	0.181
Mean Age [years] (SD)	67.4 (18.7)	69.6 (18.9)	5320	<0.001
Median Length of Stay (days) (IQR)	3.0 (5.8)	3.7 (6.1)	-	<0.001
COVID-19 (%)	343 (8.7)	277 (11.0)	1	0.003
**Mortality**				
Mortality with-in 24 hours (%)	30 (0.8)	32 (1.3)	1	0.058
Mortality with-in 48 hours (%)	61 (1.6)	48 (1.9)	1	0.335
Mortality with-in 72 hours (%)	96 (2.4)	68 (2.7)	1	0.585
In-hospital mortality	323 (8.2)	212 (8.4)	1	0.833
Mean NEWS2 (SD)	2.8 (2.8)	3.2 (2.8)	5446	<0.001
**Vital Signs**				
Mean Respiratory rate [breaths per minute] (SD)	19.8 (5.1)	20.7 (5.6)	5027	<0.001
Mean Temperature [^{o}C] (SD)	36.4 (0.9)	36.3 (1)	4817	0.001
Mean Systolic pressure [mmHg] (SD)	141.8 (29.2)	142 (28.5)	5455	0.839
Mean Diastolic pressure [mmHg] (SD)	79.2 (16.5)	79 (17.3)	5193	0.545
Mean Pulse rate [beats per minute] (SD)	89.1 (22.3)	88.5 (22.1)	5406	0.336
Mean Oxygen saturation (SD)	96.3 (3.1)	96.1 (3.2)	5182	0.059
Oxygen supplementation (%)	512 (13)	362 (14.4)	1	0.142
Mean oxygen flow rate (units) (SD)	7.1 (5.7)	6.1 (5.3)	811	0.007
Oxygen scale 2 (yes) (%)	240 (6.1)	163 (6.5)	1	0.605
**Alertness**				
Alert (%)	3510 (89.4)	2243 (89)	5	0.010
Baseline confusion (%)	27 (0.7)	23 (0.9)		
New confusion (%)	61 (1.6)	40 (1.6)		
Pain (%)	32 (0.8)	17 (0.7)		
Voice (%)	151 (3.8)	134 (5.3)		
Unconscious (%)	143 (3.6)	63 (2.5)		
Mean Albumin [g/L] (SD)	40.3 (5.7)	40.2 (5.8)	4484	0.508
Mean Creatinine [umol/L] (SD)	106.3 (104.1)	103 (82.5)	5125	0.194
Mean Haemoglobin [g/l] (SD)	126.1 (23.4)	127.5 (22.3)	4680	0.027
Mean Potassium [mmol/L] (SD)	4.4 (0.6)	4.4 (0.6)	4449	0.135
Mean Sodium [mmol/L] (SD)	138.3 (5)	137.9 (5.3)	4349	0.016
Mean White cell count [10^9 cells/L] (SD)	10.3 (7.6)	11 (5.9)	5147	<0.001
Mean Urea [mmol/L] (SD)	7.9 (6.2)	8.3 (6.6)	4382	0.017
AKI Score			2.2	0.158
0 (%)	2900 (92)	1916 (90.5)		
1 (%)	137 (4.3)	120 (5.7)		
2 (%)	61 (1.9)	46 (2.2)		
3 (%)	53 (1.7)	36 (1.7)		

Table 1 Characteristics of emergency medical admissions in development and validation datasets.

We assessed the performance of CARMc19_N and CARMc19_NB models to predict the risk of in-hospital mortality in emergency medical admissions (see Table 2 and Figure 1 for validation results and Table S3 and Figure S7 for model development results).

The c-statistics for predicting mortality for CARMc19_NB was slightly higher than Model CARMc19_N in development dataset (CARMc19_NB = 0.87 (95%CI 0.85 to 0.89) vs CARMc19_N = 0.86 (95%CI 0.84 to 0.87)) and the validation dataset (CARMc19_NB = 0.88 (95%CI 0.86 to 0.90) vs CARMc19_N = 0.86 (95%CI 0.83 to 0.88)).

The c-statistics for predicting mortality for COVID-19 patients lower than non-COVID-19 patients (CARMc19_NB: 0.78 (95%CI 0.71 to 0.84) vs 0.87 (95%CI 0.84 to 0.90); CARMc19_N: 0.75 (95%CI 0.69 to 0.81) vs 0.83 (95%CI 0.79 to 0.86)).

Model	COVID-19	Mean risk discharged alive	Mean risk discharged deceased	ARD	Scaled brier score	AUC (95% CIs)	Calibration Slope
CARMc19_N	No	0.05	0.17	0.12	0.05	0.83 (0.79 to 0.86)	1.11 (0.94 to 1.27)
CARMc19_N	Yes	0.28	0.48	0.20	0.20	0.75 (0.69 to 0.81)	0.85 (0.57 to 1.13)
CARMc19_N	All	0.07	0.29	0.22	0.20	0.86 (0.83 to 0.88)	0.95 (0.83 to 1.06)
CARMc19_NB	No	0.05	0.20	0.15	0.10	0.87 (0.84 to 0.90)	1.17 (0.99 to 1.35)
CARMc19_NB	Yes	0.27	0.49	0.22	0.24	0.78 (0.71 to 0.84)	0.93 (0.61 to 1.26)
CARMc19_NB	All	0.07	0.30	0.23	0.22	0.88 (0.86 to 0.90)	1.01 (0.88 to 1.14)

Table 2: Performance of CARMc19_N and CARMc19_NB models for predicting the risk of mortality for COVID-19 and non-COVID-19 patients in validation dataset

ARD: Absolute risk difference; AUC: Area under the curve; CIs: Confidence intervals

For peer review only

Internal validation of both models is shown in Figure S6. Both models had good internal and external calibration (CARMc19_NB: 1.01 (95%CI 0.88 vs 1.14) & CARMc19_N: 0.95 (95%CI 0.83 to 1.06)) (see Table 2 and Figure 2).

Table 3 includes the sensitivity, specificity, positive and negative predictive values for CARMc19_N and CARMc19_NB models for predicting mortality at NEWS2 threshold of 4+, 5+, 6+.

At all NEWS2 thresholds (4+, 5+, 6+) model CARMc19_NB had better sensitivity (development dataset: 76% vs 72%; 71% vs 67%; 65% vs 61% and validation dataset: 79% vs 73%; 75% vs 68%; 69% vs 61%) and similar specificity (development dataset: 81% vs 82%; 86% vs 86%; 89% vs 90% and validation dataset: 80% vs 82%; 85% vs 86%; 88% vs 89%) (see Table 3 & S4).

Model	At NEWS score [predicted risk of death]	Number of deaths identified by model	Sensitivity%	Specificity%	PPV	NPV	LR+	LR-
CARMc19_N	4+ [0.09]	696	73.1 (66.6 to 79)	81.8 (80.2 to 83.4)	27 (23.4 to 30.8)	97.1 (96.2 to 97.8)	4 (3.6 to 4.5)	0.3 (0.3 to 0.4)
CARMc19_N	5+ [0.11]	557	68.4 (61.7 to 74.6)	86 (84.5 to 87.4)	31 (26.8 to 35.4)	96.7 (95.9 to 97.5)	4.9 (4.3 to 5.6)	0.4 (0.3 to 0.4)
CARMc19_N	6+ [0.14]	452	61.8 (54.9 to 68.4)	89.1 (87.8 to 90.4)	34.3 (29.5 to 39.3)	96.2 (95.3 to 97)	5.7 (4.9 to 6.7)	0.4 (0.4 to 0.5)
CARMc19_NB	4+ [0.09]	651	79.1 (72.5 to 84.8)	79.8 (77.9 to 81.6)	26.9 (23.2 to 30.9)	97.6 (96.7 to 98.3)	3.9 (3.5 to 4.4)	0.3 (0.2 to 0.3)
CARMc19_NB	5+ [0.11]	526	75.3 (68.3 to 81.4)	84.8 (83.1 to 86.3)	31.7 (27.3 to 36.3)	97.3 (96.4 to 98)	4.9 (4.3 to 5.6)	0.3 (0.2 to 0.4)
CARMc19_NB	6+ [0.14]	431	69.2 (62 to 75.8)	88.1 (86.5 to 89.5)	35.3 (30.3 to 40.5)	96.8 (95.9 to 97.6)	5.8 (5 to 6.8)	0.3 (0.3 to 0.4)

Table 3 Sensitivity analysis of CARMc19_N and CARMc19_NB models in validation dataset for predicting the risk of mortality at NEWS2 thresholds 4+ [0.09] ,5+ [0.11], and 6+ [0.14] of predicted risk of mortality in development dataset.

PPV = Positive Predictive Value; NPV = Negative Predictive Value; LR+ = Positive Likelihood Ratio; LR- = Negative Likelihood Ratio

For peer review only

Discussion

In this study, we developed and validated two (CARMc19_N and CARMc19_NB) models to predict the risk of in-hospital mortality with the following covariates: 1) CARMc19_N uses age + sex + COVID-19 (yes/no) + NEWS2 including sub components; 2) CARMc19_NB extends Model CARMc19_N with all seven blood test results and AKI score (see appendix for equations and escalation policy Figure S5). We found that CARMc19 scores have good performance chractersttics and our findings tentatively suggest that a NEWS2 threshold of 5+ appears to strike a reasonable balance between sensitivity and specificity. CARMc19_NB was more sensitive with similar specificity than the CARMc19_N model.

CARMc19 scores performed better than our previous CARM models [6] [7] because of additional NEWS2 variables (oxygen flow rate & oxygen scale 2) and COVID-19 status. A recent systematic review identified models to predict mortality from COVID-19 with c-statistics that ranged from 0.87 to 1 [21]. However, despite these high c-statistics, the review authors cautioned against the use of these models in clinical practice because of the high risk of bias and poor reporting of studies which are likely to have led to optimistic results [21]. In contrast, our apporoach follows rigrous methodological standards for the development of risk scores [22–24].

The main advantages of our models are that they are designed to incorporate data which are already available in the patient's electronic health record thus placing no additional data collection or computational burden on clinicians, and are readily automated. Nonetheless, we emphasise that our computer-aided risk scores are not designed to replace clinical judgement. They are intended and designed to support, not subvert, the clinical decision-making process and can be always overridden by clinical concern [5,25]. The working hypothesis for our models is that they may enhance situational awareness of mortality by processing information already available without impeding the workflow of clinical staff, especially as our approach offers a faster and less expensive assessment of in-hospital mortality risk than current laboratory tests which may be more practical to use for large numbers of people.

For peer review only

There are limitations in relation to our study. We identified COVID-19 based on ICD-10 code 'U071' which was determined by COVID-19 swab test results (hospital or community) and clinical judgment and so our findings are constrained by the accuracy of these methods [26,27]. This does, however, allow the algorithm to take account of the entry of diagnostic information by the clinician including radiology findings as input variables if the swab result is negative. The systematically lower c-statistics for COVID-19 admissions requires further study. There are several candidate hypotheses which stem from the complex pathology of COVID -19 - which can produce an inflammatory response (sepsis), coagulatopathy (leading to sudden pulmonary embolism or arterial thrombosis). It is known that NEWS(2) is inadequate in monitoring hospital patients at risk of neurological deterioration, and this may also apply, to some extent, to to COVID -19. Also, COVID -19 status could has a longer 'sell by date'. A PCR test may be positive up to 90 days after the initial infection and may therefore overestimate risk, if the patient is admitted and positive, when the COVID-19 episode is effectively over. Conversely the physiological and pathological variables are unlikely to reflect the future risk if mortality is secondary to a sudden event such as veno-thromboembolism. COVID-19 diagnosis may also be determined by clinical diagnosis (as well as PCR positive test) whereas the other variables in our models are measurements (also subject to error, but less so than a diagnostic category).

We used the index NEWS2 data in our models, but vital signs and blood test results are repeatedly updated for each patient according to hospital protocols. Although we developed models using one hospital's data and validated into another hospital's data, the extent to which changes in vital signs over time reflect changes in mortality risk need to be incorporated in our models requires further study. Our two hospitals are part of the same NHS Trust and this may undermine the generalisability of our findings, which merit further external validation.

Although we focused on in-hospital mortality (because we aimed to aid clinical decision making in the hospital), the impact of this selection bias needs to be assessed by capturing out-of-hospital mortality by linking death certification data and hospital data. CARMc19, like other risk scores, can only be an

For peer review only

aid to the decision-making process of clinical teams [12,28] and its usefulness in clinical practice remains to be seen.

The next phase of this work is to field test CARMc19 scores by carefully engineering it into routine clinical practice to see if it does enhance the quality of care for acutely ill patients, whilst noting any unintended consequences.

Conclusion

We developed a validated a risk predictor (CARMc19 score) with good performance characteristics for predicting the risk of in-hospital mortality following an emergency medical admission during the pandemic where a significant proportion of the patient cohort was presenting with COVID-19 disease. Since the presentation of the CARMc19 scores to the clinician's caring for the patient placed no additional data collection burden on clinicians and is readily automated, it was carefully introduced to the electronic care record for clinicians caring for COVID-19 patients in the hospital during the second phase of the pandemic.

Competing Interests

The authors declare no conflicts of interest. All authors have completed the (available on request from the first author) and declare: no support from any organisation for the submitted work [other than the funders described below]; no financial relationships with any organisations that might have an interest in the submitted work in the previous three years; and no other relationships or activities that could appear to have influenced the submitted work.

Funding

This research was supported by the Health Foundation. The Health Foundation is an independent charity working to improve the quality of healthcare in the UK. This research was also supported by the National Institute for Health Research (NIHR) Yorkshire and Humber Patient Safety Translational Research Centre (NIHR Yorkshire and Humber PSTRC). The views expressed in this article are those of the author(s) and not necessarily those of the NHS, the Health Foundation, the NIHR, or the Department of Health.

Role of the Funding Source

For peer review only

The funders of the study had no role in study design, data collection, data analysis, data interpretation, or writing of the report.

Author Contributions

DR and MAM had the original idea for the work. KB, RH provided the data extracts. MF undertook the statistical analyses with support from MAM. MF, MAM, and DR wrote the first draft of the paper. DR provided clinical perspectives. All others contributed to the final paper and have approved the final version. DR & MF will act as study guarantors.

Transparency Declaration

The lead author (the manuscript's guarantor) affirms that the manuscript is an honest, accurate, and transparent account of the study being reported and that no important aspects of the study have been omitted.

Ethical Approval

This study was deemed to be exempt from ethical approval because it was classified as an evaluation. Furthermore, this study used already de-identified data from an ongoing study involving NEWS which received ethical approval from Health Research Authority (HRA) and Health and Care Research Wales (HCRW) (reference number 19/HRA/0548).

Patient Involvement: None.

Data Sharing: Our data sharing agreement is with York hospital and does not permit us to share the data used in this paper.

Figure 1 Receiver Operating Characteristic curve for CARMc19_N and CARMc19_NB in predicting the risk of mortality in the development dataset

Note: predicted probability at NEWS2 thresholds 4+ [0.09], 5+ [0.11], 6+ [0.14] (sensitivity, specificity)

Figure 2 External validation of CARMc19_N and CARMc19_NB models, respectively for predicting the risk of mortality

NB: We limit the risk of mortality to 0.30 for visualisation purpose because beyond this point, we have few patients.

For peer review only

References

1 Gorbalenya AE, Baker SC, Baric RS, *et al.* The species Severe acute respiratory syndrome-related coronavirus: classifying 2019-nCoV and naming it SARS-CoV-2. Nat. Microbiol. 2020;**5**:536–44. doi:10.1038/s41564-020-0695-z

2 Onder G, Rezza G, Brusaferro S. Case-Fatality Rate and Characteristics of Patients Dying in Relation to COVID-19 in Italy. JAMA - J. Am. Med. Assoc. 2020;**323**:1775–6. doi:10.1001/jama.2020.4683

3 Vincent JL, Taccone FS. Understanding pathways to death in patients with COVID-19. Lancet Respir. Med. 2020;**8**:430–2. doi:10.1016/S2213-2600(20)30165-X

4 NHS. Royal College of Physicians: NHS England approves use of National Early Warning Score (NEWS) 2 to improve detection of acutely ill patients. 2017. https://www.rcplondon.ac.uk/news/nhs-england-approves-use-national-early-warning-score-news-2-improve-detection-acutely-ill

5 Royal College of Physicians. National Early Warning Score (NEWS): Standardising the assessment of acuteillness severity in the NHS - Report of a working party. 2012.

6 Faisal M, Richardson D, Scally A, *et al.* Performance of externally validated enhanced computer-aided versions of the National Early Warning Score in predicting mortality following an emergency admission to hospital in England: A cross-sectional study. *BMJ Open* 2019;**9**. doi:10.1136/bmjopen-2019-031596

7 Faisal M, Scally A, Jackson N, *et al.* Development and validation of a novel computer-aided score to predict the risk of in-hospital mortality for acutely ill medical admissions in two acute hospitals using their first electronically recorded blood test results and vital signs: a cross-section. *BMJ Open (accepted Oct 2018)* 2018.

8 Faisal M, Khatoon B, Scally A, *et al.* A prospective study of consecutive emergency medical admissions to compare a novel automated computer-aided mortality risk score and clinical judgement of patient mortality risk. *BMJ Open* 2019;**9**:e027741. doi:10.1136/bmjopen-2018-027741

9 Palace J, Wingerchuk DM, Fujihara K, *et al.* Benefits of eculizumab in AQP4+ neuromyelitis optica spectrum disorder: Subgroup analyses of the randomized controlled phase 3 PREVENT trial. *Mult Scler Relat Disord* 2021;**47**. doi:10.1016/J.MSARD.2020.102641

10 StatCorp. Stata: Release 14. Statistical Software. College Station, TX: StataCorp LP. 2016.

11 Steyerberg EW. *Clinical Prediction Models. A practical approach to development, validation and updating.* Springer 2008.

12 Hanley JA, McNeil BJ. The meaning and use of the area under a receiver operating characteristic (ROC) curve. *Radiology* 1982;**143**:29–36. doi:10.1148/radiology.143.1.7063747

13 Robin X, Turck N, Hainard A, *et al.* pROC: an open-source package for R and S+ to analyze and compare ROC curves. *BMC Bioinformatics* 2011;**12**:77. doi:10.1186/1471-2105-12-77

14 R Development Core Team. R: A language and environment for statistical computing. R Foundation for Statistical Computing http://www.r-project.org/. 2015.

15 Steyerberg EW, Harrell FE, Borsboom GJJ., *et al.* Internal validation of predictive models: Efficiency of some procedures for logistic regression analysis. *J Clin Epidemiol* 2001;**54**:774–81. doi:10.1016/S0895-4356(01)00341-9

16 Harrell FE. rms: Regression Modeling Strategies http://cran.r-project.org/package=rms. 2015.

For peer review only

17 National Institute for Health and Care Excellence. Sepsis Quality Standard [QS161]. Published September 2017. https://www.nice.org.uk/guidance/qs161/resources/sepsis-pdf-75545595402181

18 NHS England. Sepsis Guidance implementation advice for adults: NHS England, Sept 17. https://www.england.nhs.uk/wp-content/uploads/2017/09/sepsis-guidance-implementation-advice-for-adults.pdf

19 Sing T, Sander O, Beerenwinkel N, *et al.* ROCR: visualizing classifier performance in R. *Bioinformatics* 2005;**21**:3940–1. doi:10.1093/bioinformatics/bti623

20 Moons KGM, Altman DG, Reitsma JB, *et al.* Transparent Reporting of a multivariable prediction model for Individual Prognosis Or Diagnosis (TRIPOD): Explanation and Elaboration. *Ann Intern Med* 2015;**162**:W1. doi:10.7326/M14-0698

21 Wynants L, Van Calster B, Collins GS, *et al.* Prediction models for diagnosis and prognosis of covid-19: Systematic review and critical appraisal. *BMJ* 2020;**369**:18. doi:10.1136/bmj.m1328

22 Steyerberg EW, Vergouwe Y. Towards better clinical prediction models: seven steps for development and an ABCD for validation. *Eur Heart J* 2014;**35**:1925–31. doi:10.1093/eurheartj/ehu207

23 Cowley LE, Farewell DM, Maguire S, *et al.* Methodological standards for the development and evaluation of clinical prediction rules: a review of the literature. *Diagnostic Progn Res 2019 31* 2019;**3**:1–23. doi:10.1186/S41512-019-0060-Y

24 Van Calster B, Nieboer D, Vergouwe Y, *et al.* A calibration hierarchy for risk models was defined: from utopia to empirical data. *J Clin Epidemiol* Published Online First: 6 January 2016. doi:10.1016/j.jclinepi.2015.12.005

25 Balamuth F, Alpern ER, Abbadessa MK, *et al.* Improving Recognition of Pediatric Severe Sepsis in the Emergency Department: Contributions of a Vital Sign-Based Electronic Alert and Bedside Clinician Identification. *Ann Emerg Med* 2017;**70**:759-768.e2. doi:10.1016/j.annemergmed.2017.03.019

26 Corfield AR, Lees F, Zealley I, *et al.* Utility of a single early warning score in patients with sepsis in the emergency department. 2012.

27 Churpek MM, Snyder A, Han X, *et al.* Quick Sepsis-related Organ Failure Assessment, Systemic Inflammatory Response Syndrome, and Early Warning Scores for Detecting Clinical Deterioration in Infected Patients outside the Intensive Care Unit. *Am J Respir Crit Care Med* 2017;**195**:906–11. doi:10.1164/rccm.201604-0854OC

28 Brabrand M, Folkestad L, Clausen NG, *et al.* Risk scoring systems for adults admitted to the emergency department: a systematic review. *Scand J Trauma Resusc Emerg Med* 2010;**18**:8. doi:10.1186/1757-7241-18-8

For peer review only

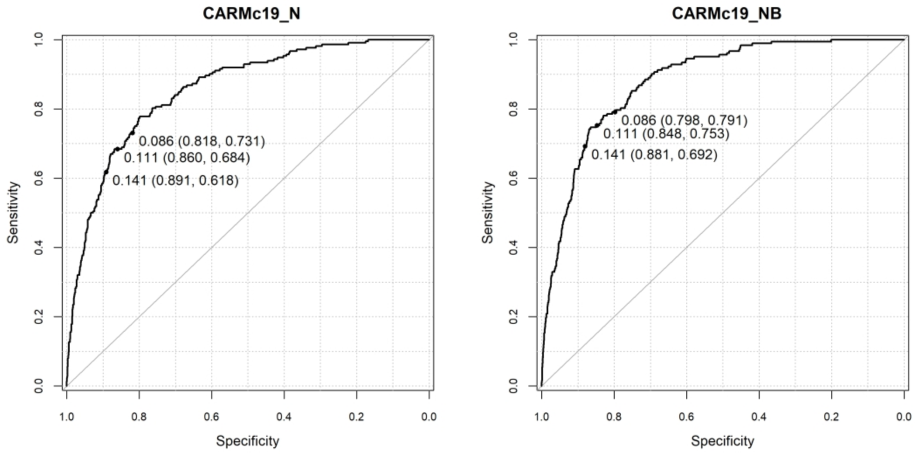

Figure 1 Receiver Operating Characteristic curve for CARMc19_N and CARMc19_NB in predicting the risk of mortality in the development dataset
Note: predicted probability at NEWS2 thresholds 4+ [0.09], 5+ [0.11], 6+ [0.14] (sensitivity, specificity)

304x152mm (300 x 300 DPI)

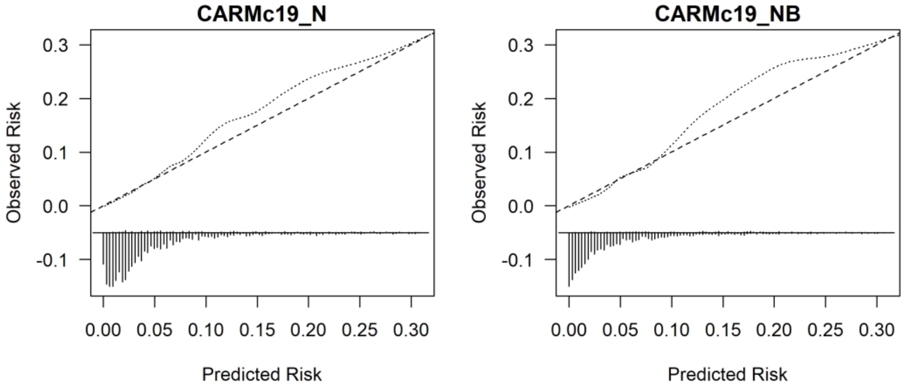

Figure 2 External validation of CARMc19_N and CARMc19_NB models, respectively for predicting the risk of mortality
NB: We limit the risk of mortality to 0.30 for visualisation purpose because beyond this point, we have few patients.

228x101mm (300 x 300 DPI)

Supplementary Material

Model CARMc19_N: predicting in-hospital mortality using vital signs only (N)

$$Logit(Died) = 10.671 + 9.262 * Male + 0.044 * Age + 0.069 * NEWS2 + 0.845 * log(Respiratory\ Rate) - 0.276 * Temperature - 0.022 * log(Diastolic\ pressure) - 1.524 * log(Systolic\ pressure) + 0.438 * log(Pulse\ rate) - 0.044 * Oxygen\ Saturations + 0.125 * Oxygen\ Supplementation + 0.487 * Baseline\ Confusion - 19.329 * New\ Confusion + 0.789 * Pain + 0.476 * Voice + 1.608 * Unconscious + 0.349 * Oxygen\ Scale\ 2 + 0.056 * Oxygen\ Flow\ Rate - 8.947 * COVID19 - 0.096 * Male * Oxygen\ Saturation + 3.856 * log(Systolic\ pressure) * New\ Confusion + 0.085 * Oxygen\ Saturation * COVID19 + 0.036 * Age * COVID19$$

Model CARMc19_NB: predicting in-hospital mortality using vital signs (N) and blood test results (B)

$$Logit(Died) = 14.241 - 1.478 * Male + 0.041 * Age - 0.063 * Albumin + 5.900 * log(Creatinine) - 0.002 * Haemoglobin - 0.830 * log(Potassium) - 0.043 * Sodium + 0.342 * log(White\ Cell\ Count) + 0.181 * log(Urea) + 0.434 * AKI\ Score\ 1 + 0.465 * AKI\ Score\ 2 + 0.406 * AKI\ Score\ 3 + 0.050 * NEWS2 + 0.729 * log(Respiratory\ Rate) - 0.257 * Temperature + 0.863 * log(Diastolic\ pressure) - 0.875 * log(Systolic\ pressure) + 0.066 * log(Pulse\ rate) - 0.055 * Oxygen\ saturation + 0.175 * Oxygen\ supplementation + 0.393 * Baseline\ Confusion - 0.414 * New\ Confusion + 1.085 * Pain + 1.024 * Voice + 2.635 * Unconscious + 0.611 * Oxygen\ Scale\ 2 + 0.026 * Oxygen\ Flow\ rate + 1.934 * COVID19 + 0.648 * Male * log(Urea) + 0.127 * New\ Confusion * log(White\ Cell\ Count)$$

We accounted for baseline difference in risk of mortality in the external validation data by subtracting (CARMc19_N: 0.24 and CARMc19_NB:0.27) from the CARMc19 logit models using an iterative procedure described elsewhere[1].

1. Faisal M, Howes R, Steyerberg EW, Richardson D, Mohammed MA. Using routine blood test results to predict the risk of death for emergency medical admissions to hospital: an external model validation study. QJM [Internet]. 2017 Jan 1 [cited 2017 Oct 2];110(1):27–31. Available from: https://academic.oup.com/qjmed/article-lookup/doi/10.1093/qjmed/hcw110

For peer review only

Table S1: NEWS2 scoring chart

Physiological Parameters	3	2	1	0	1	2	3
Respiration Rate	≤8		9 - 11	12 - 20		21 - 24	≥25
SpO2 Scale 1 (%)	≤91	92 - 93	94 - 95	≥96			
SpO2 Scale 2 (%)	≤83	84 - 85	86 - 87	88 - 92<br>≥93 on Air	93 – 94 on oxygen	95 – 96 on oxygen	≥97 on oxygen
Oxygen Saturations	≤91	92 - 93	94 - 95	≥96			
Air or Oxygen?		Oxygen		Air			
Temperature	≤35.0		35.1 - 36.0	36.1 - 38.0	38.1 - 39.0	≥39.1	
Systolic BP	≤90	91 - 100	101 - 110	111 - 219			≥220
Heart Rate	≤40		41 - 50	51-90	91 - 110	111 - 130	≥131
Level of Consciousness				Alert			Voice, Pain, Confusion, or Unconscious

The NEWS [https://www.rcplondon.ac.uk/projects/outputs/national-early-warning-score-news] is based on a scoring system in which a score is allocated to the physiological measurements of vital signs already undertaken when patients present to or are being monitored in hospital. A score is allocated to each as they are measured, the magnitude of the score reflecting how extreme the parameter varies from the norm. This score is then aggregated and uplifted for people requiring oxygen.

Characteristic	Development dataset (YH)	Validation dataset (SH)	All
	N (%)	N (%)	N (%)
**Total emergency medical discharges between 11 Mar 20 to 13 June 20**	3952	2528	6480
**Excluded: No NEWS2 recorded (%)**	13 (0.3)	6 (0.2)	19 (0.3)
**Excluded: First NEWS2 after 24 hours of admission (%)**	15 (0.4)	2 (0.1)	17 (0.3)
**Excluded: First blood test results after 4 days of admission (%)**	786 (20.0)	403 (15.9)	1189 (18.3)
**Total excluded (%)**	814 (20.6)	411 (16.3)	1225 (18.9)
**Total included (%)**	3138 (79.4)	2117 (84.7)	5255 (81.1)

Table S2 Number of emergency medical admissions included/excluded

For peer review only

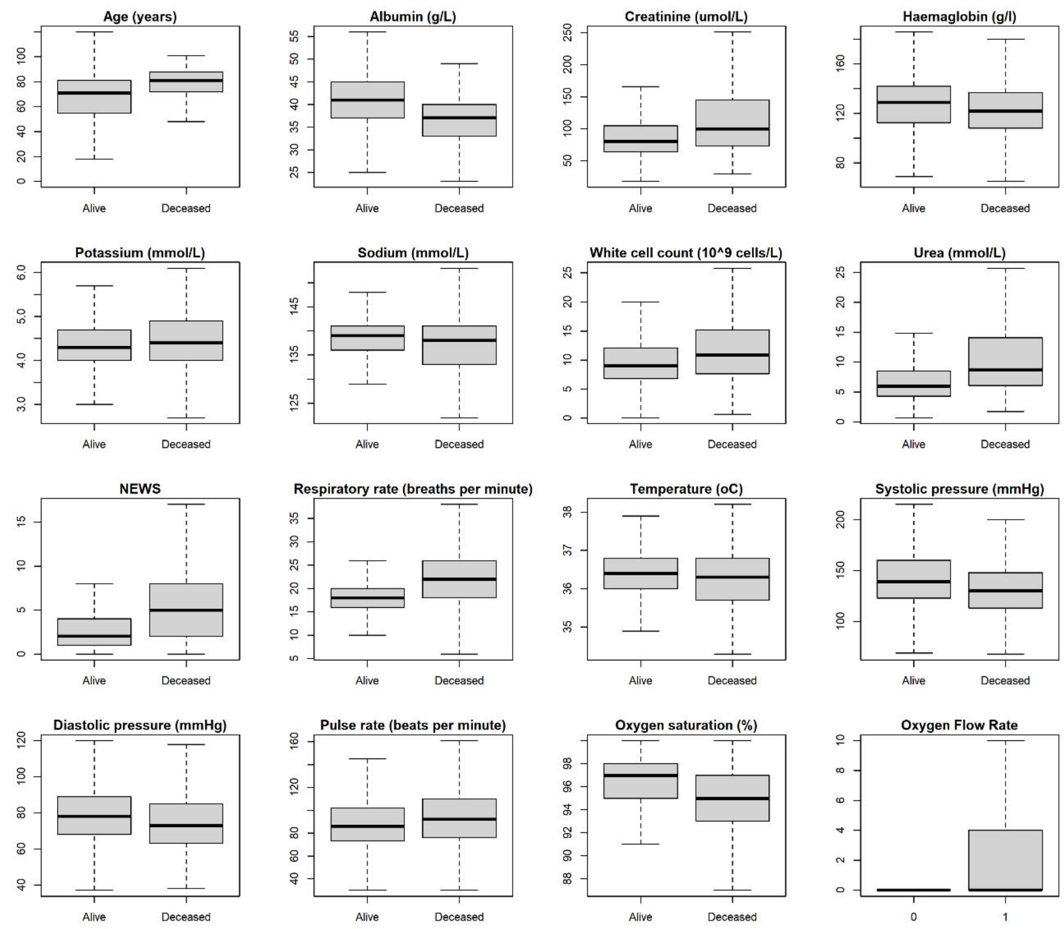

Figure S1 Boxplot for continuous covariates without outliers with respect to discharge status (alive/deceased) for development dataset.

For peer review only - http://bmjopen.bmj.com/site/about/guidelines.xhtml

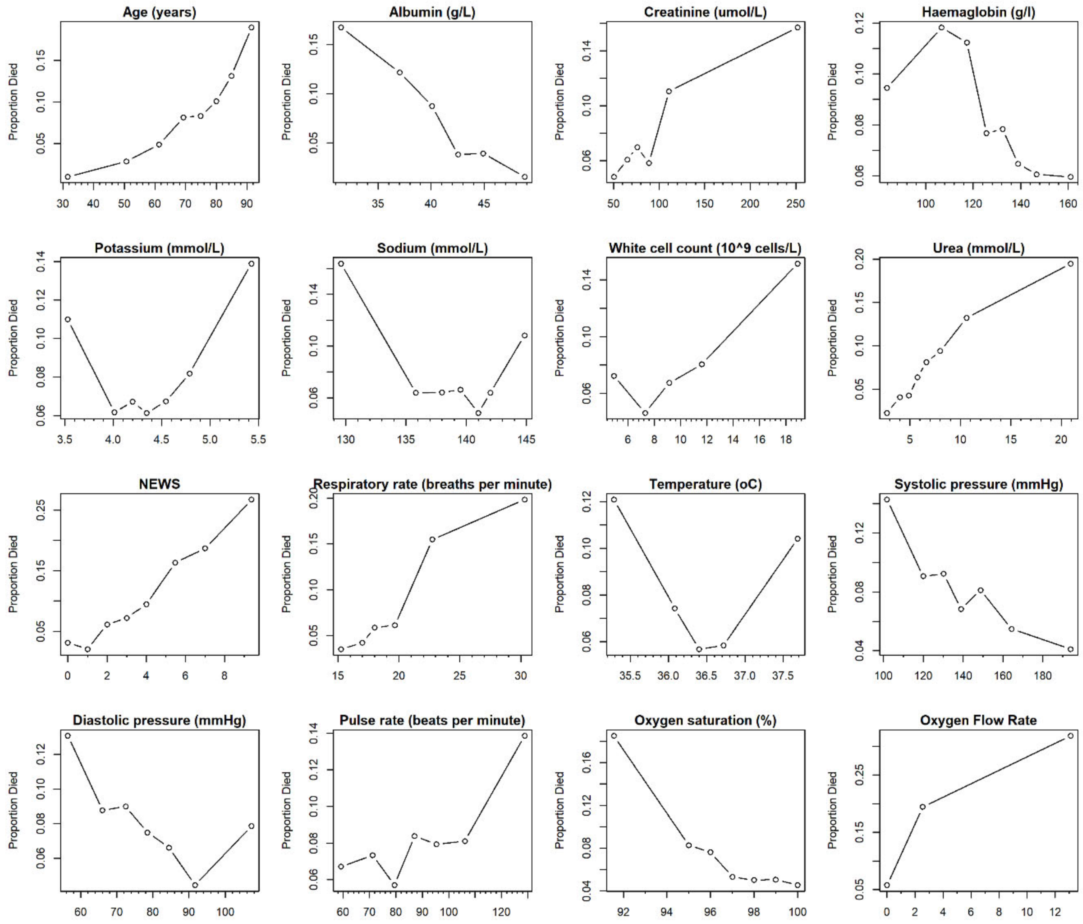

Figure S2 Scatter plots showing the observed risk of in-hospital mortality with continuous covariates for development dataset.

NB: y-axis range changes in each plot.

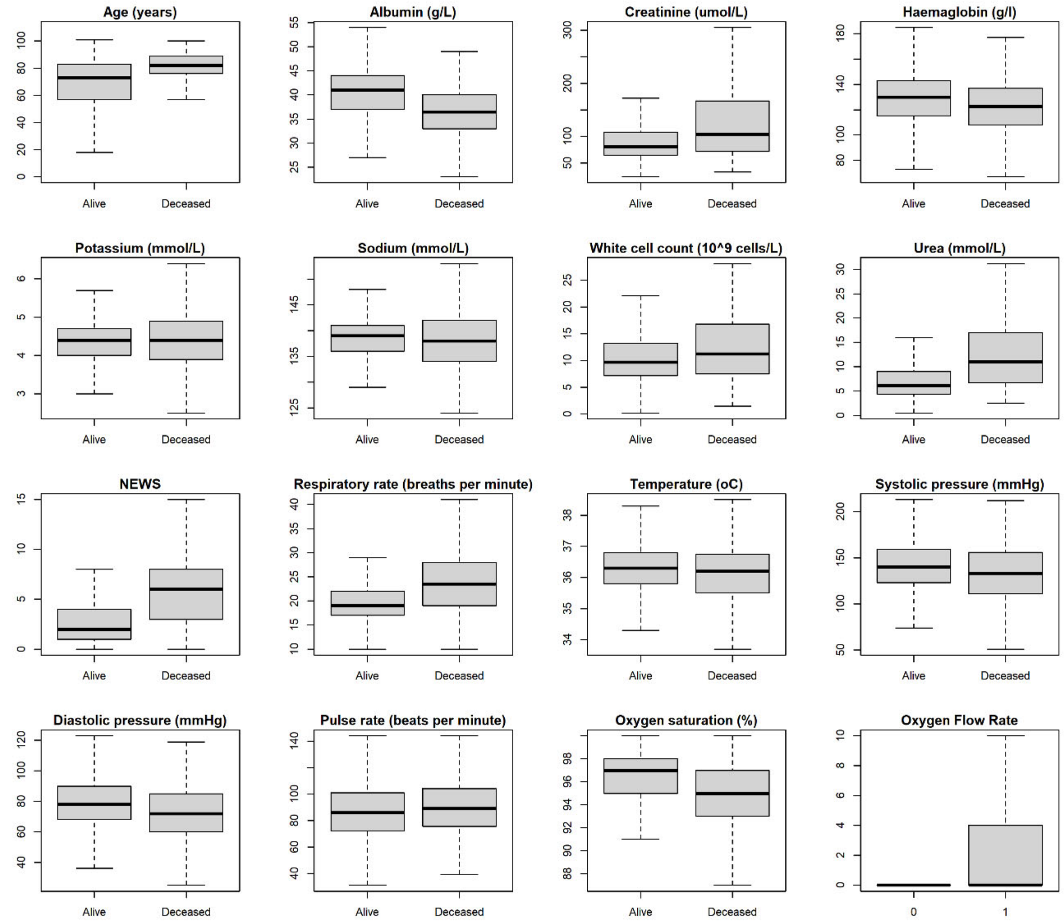

Figure S3 Boxplot for continuous covariates without outliers with respect to discharge status (alive/deceased) for validation dataset.

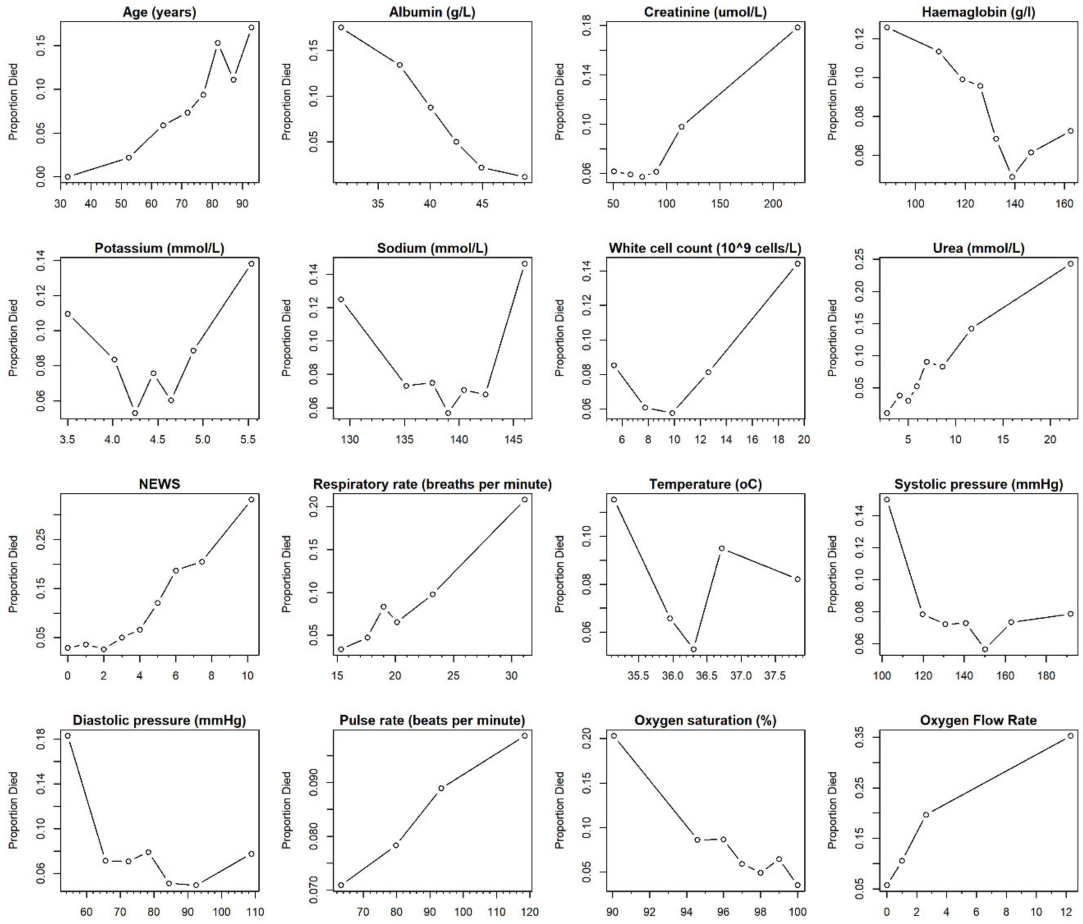

Figure S4 Scatter plots showing the observed risk of in-hospital mortality with continuous covariates for validation dataset.

NB: y-axis range changes in each plot.

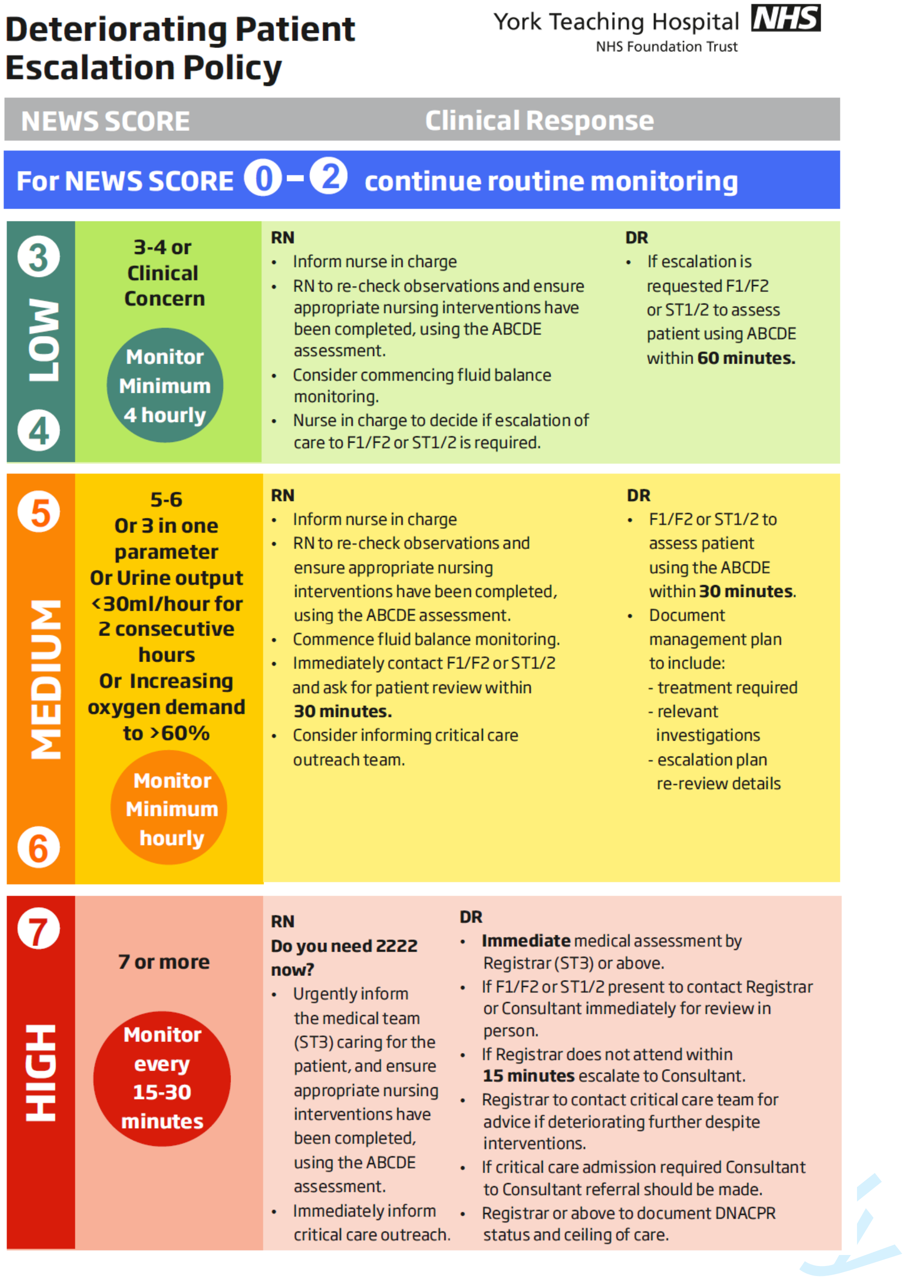

Deteriorating Patient Escalation Policy

York Teaching Hospital NHS
NHS Foundation Trust

NEWS SCORE		Clinical Response	
For NEWS SCORE 0–2 continue routine monitoring			
LOW 3 4	3-4 or Clinical Concern. Monitor Minimum 4 hourly	**RN**<br>• Inform nurse in charge<br>• RN to re-check observations and ensure appropriate nursing interventions have been completed, using the ABCDE assessment.<br>• Consider commencing fluid balance monitoring.<br>• Nurse in charge to decide if escalation of care to F1/F2 or ST1/2 is required.	**DR**<br>• If escalation is requested F1/F2 or ST1/2 to assess patient using ABCDE within **60 minutes.**
MEDIUM 5 6	5-6 Or 3 in one parameter Or Urine output <30ml/hour for 2 consecutive hours Or Increasing oxygen demand to >60%. Monitor Minimum hourly	**RN**<br>• Inform nurse in charge<br>• RN to re-check observations and ensure appropriate nursing interventions have been completed, using the ABCDE assessment.<br>• Commence fluid balance monitoring.<br>• Immediately contact F1/F2 or ST1/2 and ask for patient review within **30 minutes.**<br>• Consider informing critical care outreach team.	**DR**<br>• F1/F2 or ST1/2 to assess patient using the ABCDE within **30 minutes**.<br>• Document management plan to include:<br>- treatment required<br>- relevant investigations<br>- escalation plan re-review details
HIGH 7	7 or more. Monitor every 15-30 minutes	**RN**<br>**Do you need 2222 now?**<br>• Urgently inform the medical team (ST3) caring for the patient, and ensure appropriate nursing interventions have been completed, using the ABCDE assessment.<br>• Immediately inform critical care outreach.	**DR**<br>• **Immediate** medical assessment by Registrar (ST3) or above.<br>• If F1/F2 or ST1/2 present to contact Registrar or Consultant immediately for review in person.<br>• If Registrar does not attend within **15 minutes** escalate to Consultant.<br>• Registrar to contact critical care team for advice if deteriorating further despite interventions.<br>• If critical care admission required Consultant to Consultant referral should be made.<br>• Registrar or above to document DNACPR status and ceiling of care.

Figure S5 Escalation policy of deteriorating patients in York Teaching Hospital NHS Foundation Trust.

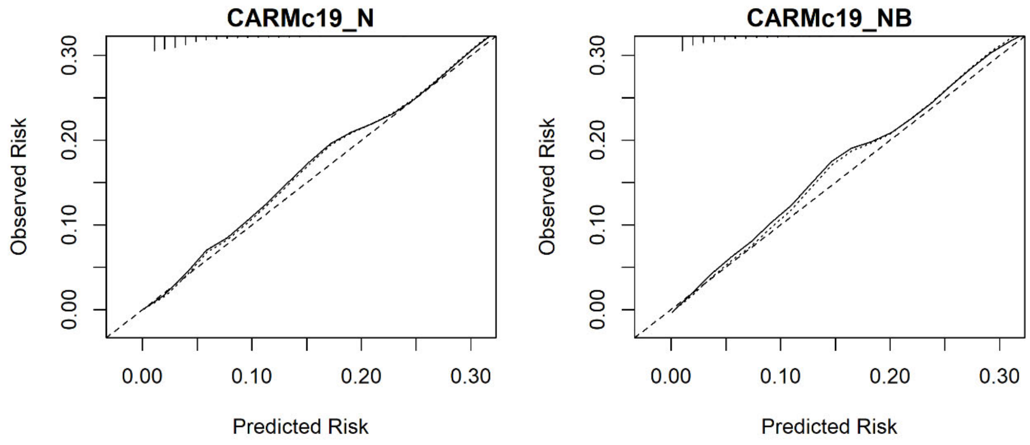

Figure S6 Calibration of CARMc19_N and CARMc19_NB models respectively for predicting the risk of mortality.

NB: We limit the risk of mortality to 0.30 for visualisation purpose because beyond this point, we have few patients.

For peer review only

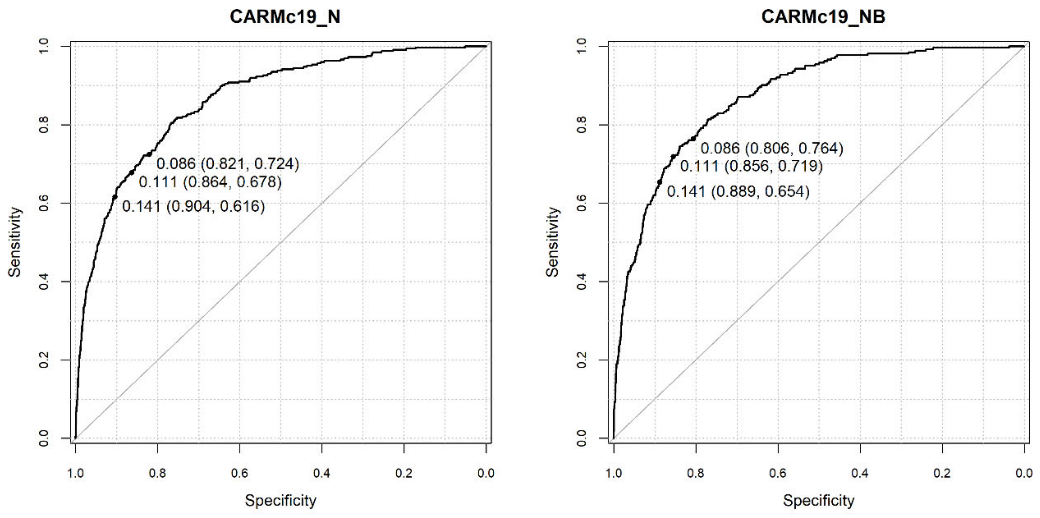

Figure S7 Receiver Operating Characteristic curve for CARMc19_N and CARMc19_NB in predicting the risk of mortality in the development dataset.

Note: predicted probability at NEWS2 thresholds 4+ [0.09], 5+[0.11], 6+[0.14] (sensitivity, specificity)

For peer review only

Model	Mean risk discharged alive	Mean risk discharged deceased	ARD	Scaled brier score	AUC (95% CIs)	Optimism-corrected c-statistic
CARMc19_N	0.06	0.31	0.25	0.24	0.86 (0.84 to 0.87)	0.85
CARMc19_NB	0.06	0.31	0.25	0.24	0.87 (0.85 to 0.89)	0.86

Table S3: Performance of CARMc19_N and CARMc19_NB models for predicting the risk of mortality in the development dataset.

ARD: Absolute risk difference; AUC: Area under the curve; CIs: Confidence intervals

Model	At NEWS score [predicted risk of death]	Number of deaths identified by the model	Sensitivity%	Specificity%	PPV	NPV	LR+	LR-
CARMc19_N	4+ [0.09]	880	72.4 (67.2 to 77.2)	82.1 (80.8 to 83.3)	26.6 (23.7 to 29.6)	97.1 (96.4 to 97.6)	4 (3.7 to 4.4)	0.3 (0.3 to 0.4)
CARMc19_N	5+ [0.11]	710	67.8 (62.4 to 72.9)	86.4 (85.2 to 87.5)	30.8 (27.5 to 34.4)	96.8 (96.1 to 97.3)	5 (4.4 to 5.6)	0.4 (0.3 to 0.4)
CARMc19_N	6+ [0.14]	544	61.6 (56.1 to 66.9)	90.4 (89.4 to 91.4)	36.6 (32.5 to 40.8)	96.3 (95.6 to 96.9)	6.4 (5.6 to 7.3)	0.4 (0.4 to 0.5)
CARMc19_NB	4+ [0.09]	750	76.4 (70.8 to 81.4)	80.6 (79.1 to 82)	26.4 (23.3 to 29.7)	97.4 (96.7 to 98)	3.9 (3.6 to 4.4)	0.3 (0.2 to 0.4)
CARMc19_NB	5+ [0.11]	602	71.9 (66 to 77.2)	85.6 (84.2 to 86.8)	31.2 (27.5 to 35)	97.1 (96.4 to 97.7)	5 (4.4 to 5.6)	0.3 (0.3 to 0.4)
CARMc19_NB	6+ [0.14]	496	65.4 (59.3 to 71.1)	88.9 (87.6 to 90)	34.8 (30.6 to 39.2)	96.6 (95.8 to 97.2)	5.9 (5.1 to 6.7)	0.4 (0.3 to 0.5)

Table S4 Sensitivity analysis of CARMc19_N and CARMc19_NB models for predicting the risk of mortality at NEWS2 thresholds (4,5,6) for development dataset.

PPV = Positive Predictive Value; NPV = Negative Predictive Value; LR+ = Positive Likelihood Ratio; LR- = Negative Likelihood Ratio

For peer review only

TRIPOD Checklist: Prediction Model Development and Validation

Section/Topic	Item		Checklist Item	Page
**Title and abstract**				
Title	1	D;V	Identify the study as developing and/or validating a multivariable prediction model, the target population, and the outcome to be predicted.	1
Abstract	2	D;V	Provide a summary of objectives, study design, setting, participants, sample size, predictors, outcome, statistical analysis, results, and conclusions.	3
**Introduction**				
Background and objectives	3a	D;V	Explain the medical context (including whether diagnostic or prognostic) and rationale for developing or validating the multivariable prediction model, including references to existing models.	5
	3b	D;V	Specify the objectives, including whether the study describes the development or validation of the model or both.	6
**Methods**				
Source of data	4a	D;V	Describe the study design or source of data (e.g., randomized trial, cohort, or registry data), separately for the development and validation data sets, if applicable.	6
	4b	D;V	Specify the key study dates, including start of accrual; end of accrual; and, if applicable, end of follow-up.	7
Participants	5a	D;V	Specify key elements of the study setting (e.g., primary care, secondary care, general population) including number and location of centres.	7
	5b	D;V	Describe eligibility criteria for participants.	7
	5c	D;V	Give details of treatments received, if relevant.	
Outcome	6a	D;V	Clearly define the outcome that is predicted by the prediction model, including how and when assessed.	8
	6b	D;V	Report any actions to blind assessment of the outcome to be predicted.	
Predictors	7a	D;V	Clearly define all predictors used in developing or validating the multivariable prediction model, including how and when they were measured.	7
	7b	D;V	Report any actions to blind assessment of predictors for the outcome and other predictors.	
Sample size	8	D;V	Explain how the study size was arrived at.	7
Missing data	9	D;V	Describe how missing data were handled (e.g., complete-case analysis, single imputation, multiple imputation) with details of any imputation method.	7
Statistical analysis methods	10a	D	Describe how predictors were handled in the analyses.	8
	10b	D	Specify type of model, all model-building procedures (including any predictor selection), and method for internal validation.	8
	10c	V	For validation, describe how the predictions were calculated.	8
	10d	D;V	Specify all measures used to assess model performance and, if relevant, to compare multiple models.	8,9
	10e	V	Describe any model updating (e.g., recalibration) arising from the validation, if done.	suppl
Risk groups	11	D;V	Provide details on how risk groups were created, if done.	
Development vs. validation	12	V	For validation, identify any differences from the development data in setting, eligibility criteria, outcome, and predictors.	8,9
**Results**				
Participants	13a	D;V	Describe the flow of participants through the study, including the number of participants with and without the outcome and, if applicable, a summary of the follow-up time. A diagram may be helpful.	10
	13b	D;V	Describe the characteristics of the participants (basic demographics, clinical features, available predictors), including the number of participants with missing data for predictors and outcome.	10,11
	13c	V	For validation, show a comparison with the development data of the distribution of important variables (demographics, predictors and outcome).	11
Model development	14a	D	Specify the number of participants and outcome events in each analysis.	10
	14b	D	If done, report the unadjusted association between each candidate predictor and outcome.	
Model specification	15a	D	Present the full prediction model to allow predictions for individuals (i.e., all regression coefficients, and model intercept or baseline survival at a given time point).	suppl
	15b	D	Explain how to the use the prediction model.	suppl
Model performance	16	D;V	Report performance measures (with CIs) for the prediction model.	11
Model-updating	17	V	If done, report the results from any model updating (i.e., model specification, model performance).	
**Discussion**				
Limitations	18	D;V	Discuss any limitations of the study (such as nonrepresentative sample, few events per predictor, missing data).	12
Interpretation	19a	V	For validation, discuss the results with reference to performance in the development data, and any other validation data.	12
	19b	D;V	Give an overall interpretation of the results, considering objectives, limitations, results from similar studies, and other relevant evidence.	12,13
Implications	20	D;V	Discuss the potential clinical use of the model and implications for future research.	13
**Other information**				
Supplementary information	21	D;V	Provide information about the availability of supplementary resources, such as study protocol, Web calculator, and data sets.	suppl
Funding	22	D;V	Give the source of funding and the role of the funders for the present study.	14

*Items relevant only to the development of a prediction model are denoted by D, items relating solely to a validation of a prediction model are denoted by V, and items relating to both are denoted D;V. We recommend using the TRIPOD Checklist in conjunction with the TRIPOD Explanation and Elaboration document.

For peer review only - http://bmjopen.bmj.com/site/about/guidelines.xhtml
